# Spatial interactions of immune cells as potential predictors to efficacy of toripalimab plus chemotherapy in locally advanced or metastatic pancreatic ductal adenocarcinoma: a phase Ib/II trial

**DOI:** 10.1038/s41392-024-02031-8

**Published:** 2024-11-25

**Authors:** Ke Cheng, Xiaoying Li, Wanrui Lv, Gang Zhao, Ruihan Zhou, Chen Chang, Heqi Yang, Ruizhen Li, Zhiping Li, Ye Chen, Cheng Yi, Ouying Yan, Chaoxin Xiao, Yi Zhang, Junjie Xiong, Zixin Huang, Weikang Shao, Xin You, Wenhao Guo, Du He, Wenwu Ling, Rui Wang, Bole Tian, Chengjian Zhao, Dan Cao

**Affiliations:** 1 Division of Abdominal Tumor, Department of Medical Oncology, Cancer Center and State Key Laboratory of Biological Therapy, West China Hospital, Sichuan University, Chengdu, Sichuan China; 2 Department of Oncology, Meishan City People’s Hospital, Meishan, Sichuan China; 3 State Key Laboratory of Biotherapy and Cancer Center, Sichuan University and Collaborative Innovation Center, West China Hospital, Chengdu, Sichuan China; 4 Pancreatic Division, Department of General Surgery, West China Hospital, Sichuan University, Chengdu, Sichuan China; 5 Department of General Surgery, ChengDu ShangJing NanFu Hospital, Chengdu, Sichuan China; 6 Department of Radiology, West China Hospital, Sichuan University, Chengdu, Sichuan China; 7 Department of Radiology, West China Tianfu Hospital, Sichuan University, Chengdu, Sichuan China; 8 Genecast Biotechnology Co. Ltd, Wuxi, China; 9 Department of Pathology, West China Hospital, Sichuan University, Chengdu, Sichuan China; 10 Department of Medical Ultrasound, West China Hospital of Sichuan University, Chengdu, Sichuan China; 11 Department of Gastroenterology, West China Hospital, Sichuan University, Chengdu, Sichuan China

**Keywords:** Cancer microenvironment, Predictive markers

## Abstract

Advanced pancreatic ductal adenocarcinoma (PDAC) has a dismal prognosis. Immunotherapy alone offers limited efficacy, but it is still unknown whether its combination with chemotherapy could offer synergistic anti-tumor effects. This phase Ib/II study evaluated the safety and efficacy of combining toripalimab with the gemcitabine plus nab-paclitaxel (GnP) regimen as first-line treatment for locally advanced or metastatic PDAC and explored predictive biomarkers (ChiCTR2000032293). The primary endpoints were safety and overall survival (OS). The secondary outcomes were objective response rate (ORR), disease control rate (DCR), and progression-free survival (PFS). Immune-related biomarkers including programmed death-ligand 1 (PD-L1) expression, genetic status, cytokine levels, and spatial features of the tumor immune microenviroment (TIME) were investigated. Neither serious treatment-related adverse events nor grade 4 immune-related adverse events were reported. Among the 72 patients, the median OS was 8.9 months, 12-month OS rate was 31.9%, with median PFS of 5.6 months, ORR of 33.3%, and DCR of 90.3%. Higher PD-L1 expression, without liver metastases were associated with higher ORR, however these factors could not effectively distinguish responders and non-responders. Importantly, dendritic cells - T helper cells - cytotoxic T lymphocytes (DC-Th-CTL) enriched immune niche and their spatial interactions were dominant predictors of response based on TIME analysis using a cyclic multiplex tissue staining assay, with an area under the curve value of 0.8. Overall, GnP plus toripalimab exhibited good safety and differentiated efficacy in selected population, and the spatial interactions of DC-Th-CTL represent promising predictors to efficacy of immunochemotherapy in locally advanced or metastatic PDAC.

## Introduction

Due to the combination of projected changes in demographics and incidence rates, pancreatic ductal adenocarcinoma (PDAC) is predicted to become to the second leading cause of cancer-related death in the United States by 2030^1^. PDAC, a highly lethal malignancy, is associated with a dismal 5-year survival rate less than 10%^2^. In current guidelines, chemotherapy has a prominent role in the comprehensive management of locally advanced or metastatic PDAC^3^. The most commonly recommended regimens include gemcitabine plus nab-paclitaxel (GnP) and FOLFIRINOX (leucovorin, 5-fluorouracil, irinotecan, and oxaliplatin)^4,5^. Despite some modest improvements in patient survival, the treatment landscape for PDAC has remained stagnant for nearly a decade, highlighting the dire need for novel treatment strategies.

Immunotherapy, especially immune checkpoint inhibitor (ICI) therapy targeting programmed cell death protein 1 (PD-1) and PD-1 ligand (PD-L1), has revolutionized the management of certain chemotherapy-resistant malignancies^6–8^. Although advanced PDAC cases with a high tumor mutational burden (TMB) and/or mismatch repair deficiency (dMMR) have shown improved responses to monotherapy with ICIs^9–11^, the efficacy of immunotherapy in PDAC with proficient mismatch repair deficiency (pMMR) accounting for 99% in all cases remains limited due to the immunosuppressive nature of the pancreatic tumor microenvironment (TME)^12,13^. Consequently, recent studies have focused on combining immunotherapy with other modalities to achieve synergistic anti-tumor effects^14,15^. Clinical trials have demonstrated the preliminary efficacy and manageable toxicity of combining anti-PD-1 antibodies with the GnP regimen. A phase Ib/II study indicated that GnP chemotherapy and pembrolizumab as a first-line treatment for patients with metastatic PDAC significantly extended progression-free survival (PFS) and overall survival (OS) to 9.1 and 15.0 months, respectively. Notably, that study reported enhanced survival benefits in patients with higher tumor cell-free DNA copy number instability^16^. A phase I clinical study indicated that combining the GnP regimen with nivolumab provided manageable safety. Biomarker analyses revealed a correlation between the treatment response and serum cytokine levels, as well as the predictive value of CD8^+^ T cell infiltration^17^. In addition, the CCTG PA.7 phase II trial investigated the combination of GnP plus durvalumab and tremelimumab. Although this combination treatment did not significantly improve OS compared to chemotherapy alone, analysis of baseline circulating tumor DNA (ctDNA) uncovered improved survival outcomes in patients with KRAS wild-type tumors^18^. Therefore, the identification of biomarkers for predicting the response to immunotherapy and combination chemotherapy in PDAC patients remains a critical need.

Toripalimab, a humanized anti-PD-1 IgG4 monoclonal antibody, has exhibited promising anti-tumor effectiveness in melanoma, lung cancer, hepatobiliary and pancreatic tumors, and other cancer types^19^. Accordingly, we conducted a phase Ib/II study to evaluate the safety and efficacy of combining toripalimab with the GnP regimen as a first-line treatment for patients with locally advanced or metastatic PDAC (mPDAC). Based on our previously reported analysis data, we completed long-term follow-up^20^. We also explored a variety of prespecified predictive biomarkers via analysis of immune microenvironment to determine whether any could identify PDAC patients who were likely to benefit from the experimental regimen.

## Results

### Patient characteristics

From April 26, 2020 to September 08, 2022, in the phase Ib study, OS beyond 1 year was observed in 10 of 18 eligible patients, corresponding to a 1-year OS rate of 55.6%. Thus, 54 eligible patients were then enrolled in the phase II study (72 cases in total) between April 26, 2020, and January 25, 2021. Efficacy and safety were evaluated in all 72 eligible patients who received at least one cycle of toripalimab in combination with the GnP regimen (Supplementary Fig. S1a). The median follow-up duration was 9.25 months at the cut-off date of April 30, 2024. The median numbers of chemotherapy cycles and immunotherapy cycles were 6 (range, 1–34 cycles) and 5.5 (range, 1–37 cycles), respectively. At the end of the study, 66 (91.7%) patients were dead, 10 patients (13.9%) had received maintenance immunotherapy, and 4 (5.6%) were still receiving treatment (Supplementary Fig. S2a). After progression, thirty-seven (37/72, 51.4%) patients underwent subsequent second-line therapy (mainly with the SOX regimen). Eleven (11/72, 15.3%) patients had received third-line therapy. One patient had entered fourth-line therapy. Detailed information regarding subsequent treatment is provided in Supplementary Fig. S2b.

At the time of enrollment, the median patient age was 57 (range, 43–71) years with a slight male-predominance (*n* = 38, 52.8%). Half of the patients had primary tumors located in the head or body/tail of the pancreas. Twenty (27.8%) of patients had an ECOG performance score of 0. Twenty (27.8%) patients had previously undergone curative pancreatectomy prior to recurrence. Forty-three (59.7%) patients had tumors at three or more sites (Supplementary Fig. S1d). The sites with the highest frequencies of metastasis were the celiac lymph nodes (*n* = 59, 81.9%) and the liver (*n* = 51, 70.8%; Supplementary Fig. S1c). Fifty-six patients (77.8%) had an elevated level of CA19-9 (CA19-9, ≥37 U/mL). The detailed baseline patient characteristics are presented in Table 1.

**Table 1:** Demographics and baseline clinical characteristics

Characteristic	No. (%)
Median age (range), y	57 (43–71)
<50	9 (12.5)
50–59	36 (50)
≥60	27 (37.5)
Sex	
male	38 (52.8)
female	34 (47.2)
BMI Median (range) (Kg/m^2^)	20.57 (15.08–33.78)
<18.5 Kg/m^2^	14 (19.4)
18.5 Kg/m^2^–23.9 Kg/m^2^	45 (62.5)
≥24 Kg/m^2^	13 (18.1)
Histologic findings	
Pancreatic ductal adenocarcinoma	72 (100)
Other	0 (0)
Location of primary tumor	
Head	36 (50)
Body/Tail	36 (50)
ECOG performance status	
0	20 (27.8)
1	51 (70.8)
2	1 (1.4)
Extent of disease	
locally advanced	6 (8.3)
metastatic	66 (91.7)
Surgery on primary tumor	
Yes	20 (27.8)
No	52 (72.2)
No. of lesion sites	
1	3 (4.2)
2	26 (36.1)
3	30 (41.7)
4	12 (16.7)
5	1 (1.4)
Sites of lesion	198
Liver	51 (70.8)
Lung	5 (6.9)
Peritoneum	24 (33.3)
Pancreas	55 (76.4)
Celiac lymph nodes	59 (81.9)
Other	4 (5.6)
CA199, median (IQR)/(Range), U/mL	403 (43.63–2827)/(0–387646)
CEA, median (IQR)/(Range), ng/mL	4.27 (2.20–9.43)/(0.3–327)
CA125, median (IQR)/(Range), U/mL	73.35 (24.6–191)/(4.83–2922)
Albumin, median (IQR)/(Range), g/L	42.45 (39.65–45)/(30.7–50.8)
<35	3 (4.2)
≥35	69 (95.8)
NLR, median (IQR)/(Range)	2.45 (1.93–3.75)/(0.84–22.56)
Bilirubin, median (IQR)/(Range)	10.75 (7.93–15.2)/(4.2–33)
≤25	69 (95.8)
>25	3 (4.2)
Hemoglobin, median (IQR)/(Range), g/L	124.5 (112–135.75)/(80–156)
Anemia	23 (31.9)
Non-anemia	49 (68.1)
Platelet, median (IQR)/(Range), 10^9/L	191 (135.75–249)/(70–519)
>300	8 (11.1)
≤300	64 (88.9)
LDH, median (IQR)/(Range), IU/L	171.5 (144.25–195.75)/(109–795)
Elevated LDH	6 (8.3)
Without elevated LDH	66 (91.7)
MMR status	
MMR-proficient tumors	71 (98.6)
MMR-deficient tumors	1 (1.4)
PD-L1 expression status	
CPS < 5	40 (55.6)
CPS ≥ 5	16 (22.2)
Unkown	16 (22.2)
TMB, median (IQR)/(Range), Muts/Mb	2.92 (1.50–4.87)/(0–70.9)
TMB-H	28 (38.9)
TMB-L	27 (37.5)
Unkown	17 (23.6)
Kras mutation status	
Kras mutation type	56 (77.8)
Kras wild type	9 (12.5)
Unknown	7 (9.7)

Data were number of patients (%), range or median (Interquartile range, IQR)

*BMI* body mass index, *ECOG* Eastern Cooperative Oncology Group, *CA199* carbohydrate antigen 19-9, *CEA* carcinoembryonic antigen, *CA125* cancer antigen 125, *NLR* neutrophil-to-lymphocyte ratio, *LDH* lactate dehydrogenase, *MMR* mismatch repair, *PD-L1* programmed cell death ligand 1, *TMB* tumor mutational burden

### Safety

All patients (*n* = 72) experienced treatment-related adverse events (TRAEs). The most common TRAEs were anemia (91.7%), hypoproteinemia (77.8%), elevated aspartate aminotransferase (69.4%), elevated alanine transaminase (66.7%), hypothyroidism (58.3%), leukopenia (52.8%), diarrhea (51.4%), neutropenia (51.4%), hyponatremia (44.4%), and anorexia (43.1%). Grade 3/4 TRAEs occurred in 38 (52.8%) patients, with the most common being neutropenia (15.3%), anemia (13.9%), leukopenia (8.3%), elevated alanine transaminase (8.3%), and elevated aspartate aminotransferase (6.9%). Five (6.9%) patients had a dose reduction of albumin-bound paclitaxel or gemcitabine due to TRAEs, and no serious treatment-related adverse event (TRAE) recurred after dose reduction (Supplementary Table S1).

Immune-related adverse events (irAEs, possibly or definitely related) were mainly grade 1/2. The most common irAEs were hypothyroidism (58.3%), rash (36.1%), myocardial enzyme elevation (11.1%), central neurotoxicity (5.6%), and interstitial pneumonia (4.2%). Five cases developed grade 3 irAEs (2 cases of myocardial enzyme elevation, 1 case of rash, 1 case of central neurotoxicity, and 1 case of interstitial pneumonia), and toripalimab was stopped in these cases. No grade 4 irAEs or treatment-related deaths were observed (Supplementary Table S1).

### Treatment efficacy

**Fig. 1:**
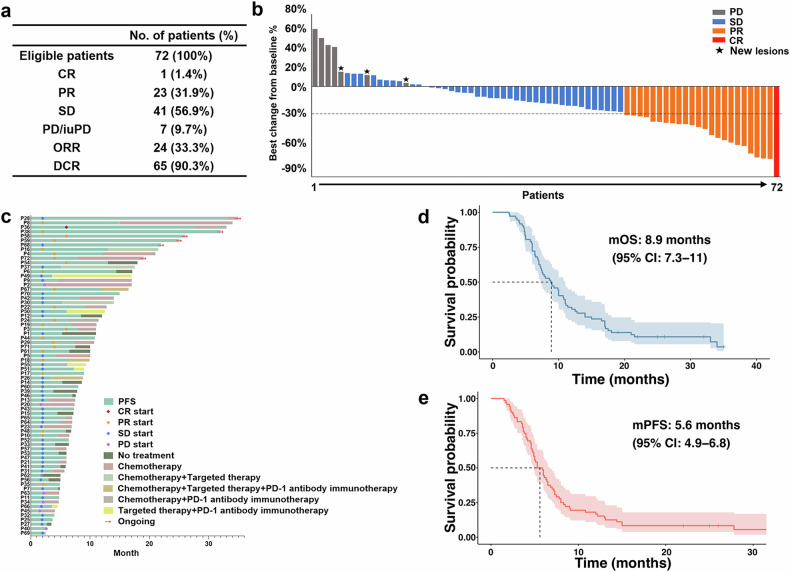
Efficacy evaluation. **a** Tumor responses were evaluated according to the Solid Tumor Response Evaluation version 1.1 standard among the intention-to-treat (ITT) population. Efficacy outcomes (CR, PR, SD, PD/iuPD, ORR and DCR) are listed. CR, complete response; PR, partial response; SD, stable disease; PD, progressive disease; iuPD, immune unconfirmed progressive disease; ORR, overall response rate (included CR and PR); DCR, disease control rate (included CR, PR, and SD). **b** Waterfall plot for treatment response. Best overall response and maximum percentage of tumor change in target lesion diameter from baseline are exhibited, based on central review of patients (*n* = 72); each bar represents one patient in the study, and is colored according to best overall response. The sum of the maximum diameters of target lesions did not increase by 20% in three patients (pentagram on top), but new lesions appeared. The sum of the maximum diameters of target lesions increased by more than 50% in one patient (leftmost gray bar). One case showed CR (rightmost red bar). **c** Swimmer’s plot showing the progression-free survival (PFS) of all participants (*n* = 72) and their following treatment type after first-line therapy, with colored rhomboid representing the timepoint of different response evalution. The length of the green bars represents the duration from enrollment to withdrawal from first-line treatment. The length of the different colored bars represents the duration of subsequent treatment or survival for each patient. For patients who are still alive, the bars end with a red arrow labeled “Ongoing”. **d**–**e** Progression-free survival and overall survival curves for the entire ITT population (*n* = 72) using Kaplan–Meier estimation. The blue and red shaded areas show the 95% confidence intervals (CI) for the overall survival (OS) and PFS curves, respectively. The dashed lines show the median PFS and median Os

Efficacy analyses were conducted among the intent-to-treat (ITT) population (*n* = 72). Complete response (CR) and partial response (PR) were achieved by 1 (1.4%) and 23 (31.9%) patients, respectively, while 41 (56.9%) patients had stable disease (SD). Progressive disease (PD) was only observed in 7 (9.7%) patients. The objective response rate (ORR) was 33.3% (24/72), and the disease control rate (DCR) was 90.3% (65/72; Fig. 1a). The individual treatment responses are shown in Fig. 1b, c. The rates of surgical conversion and R0 resection were 6.9% (5/72) and 2.8% (2/72), respectively. The median PFS was 5.6 months (95%CI: 4.9–6.8 months) (Fig. 1e), and the 6-month PFS rate was 48.6%. The median OS was 8.9 months (95%CI: 7.3–11.0 months) (Fig. 1d), and the 1-year OS rate was 31.9%. In addition, the median duration of response (DoR) was 6.2 months (95%CI: 4.3–9.2 months). The results for individual treatment response and survival time are presented in Fig. 1c. Of the 65 (90.3%) patients with available CA19-9 measurements, 73.8% (48/65) showed a decrease from baseline of at least 20%, and 26.2% (17/65) had a decrease of at least 90%. Patients with decreases in the CA19-9 level of at least 20% and 90% had a median OS duration of 9.3 and 10.0 months, respectively.

### Subgroup analysis based on clinical characteristics

The exploratory subgroup analysis found that the patients without liver metastasis had higher ORR compared with patients with liver metastasis (52.4% vs 25.5%, *P* = 0.03), and no significant survival benefit related to age; gender; body mass index (BMI); surgery on primary tumor; location of primary tumor; baseline tumor marker levels (CA 19-9 or CA125); baseline platelet, hemoglobin, or bilirubin levels; or baseline neutrophil to lymphocyte ratio (NLR). Univariate regression analysis confirmed that patients with an ECOG performance score of 0, fewer than three lesion sites, lymph node-only metastasis, an objective response (CR or PR), or without liver metastasis were significant predictors for longer median PFS and OS (Supplementary Fig. S3). A baseline albumin level ≥35 g/L, carcinoembryonic antigen (CEA) levels below median (4.27 U/ml), and locally advanced disease, separately, was associated with a better PFS, and normal lactate dehydrogenase (LDH) levels was associated with a better OS (Supplementary Fig. S3). Factors identified as statistically significant on univariate analysis were included in the multivariate analysis. Liver metastasis status, chemotherapy cycles and maintenance treatment were independent predictors of PFS, while independent predictors of OS included the baseline CA 19-9 level and maintenance treatment in multivariate regression analysis (Supplementary Tables S2 and S3). Generally, patients without liver metastasis had a better PFS and ORR than those with liver metastasis, and two representative cases achieving long PFS and OS were presented in Supplementary Fig. S4a.

### Predictive biomarkers

#### Gene mutations

**Fig. 2:**
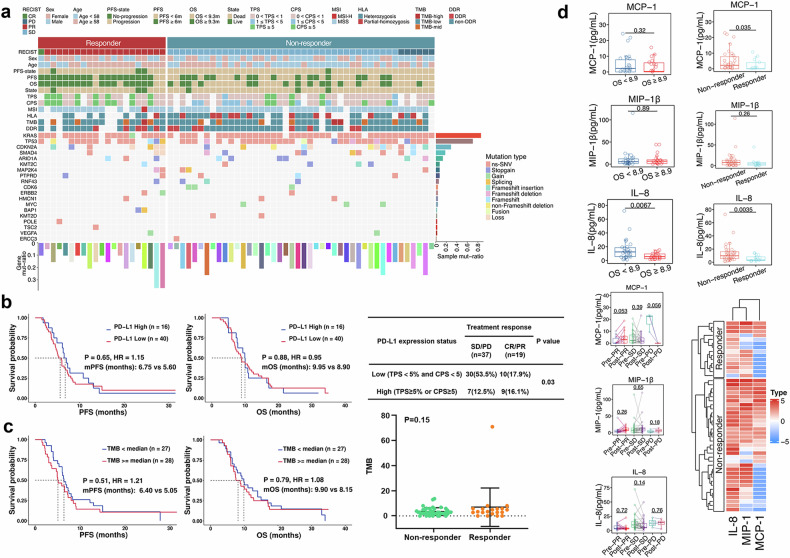
Genomic features, PD-L1 expression and serum cytokines of patients. **a** Mutational landscape of patients according to different treatment responses was exhibited. **b** Kaplan–Meier curves for PFS and OS in patients with different PD-L1 expression status, and P values are for comparisons between PD-L1 high expression and PD-L1 low expression groups using log-rank test. Hazard ratios (HR) were computed using the Cox proportional hazards model. The correlation between PD-L1 expression status and treatment response was tested using Chi-square test. **c** Kaplan–Meier curves for PFS and OS in patients with different tumor mutational burden (TMB) levels. The TMB values between non-response and response groups were compared using t-test, and no significant difference was observed. **d** Serum cytokine levels and their association with different responses and outcomes. The change of serum IL-8, MCP-1 and MIP-1β levels at baseline and post-treatment was calculated and compared according different efficacy outcomes (PR, SD and PD). Baseline serum IL-8, MCP-1, and MIP-1β levels were compared between the treatment response and non-response groups, and between in patients with long-term survial duration (OS ≥ 8.9 months) and short-term survial duration (OS < 8.9 months). Heatmap for serum IL-8, MCP-1 and MIP-1β levels displays the differences between responders and non-responders. Boxplot of the detected values for three cytokines (IL-8, MCP-1 and MIP-1β)

Patients who enrolled in the study before receiving treatment provided samples for the next-generation sequencing (NGS) analysis using the 769 cancers gene-targeted panel. Gene mutation signatures were available for 65 patients, and the most frequent mutations ranked as follows: *KRAS* (86%), *TP53* (71%), *CDKN2A* (32%), and *SMAD4* (18%) (Fig. 2a). However, these mutations were not related to the ORR, PFS and OS of the patients. In particular, we identified one patient with a *POLE* mutation, who achieved a deep response and R0 resection. We also assessed the association of DNA damage repair (DDR) gene mutations (*TP53* not included) with responses, and the landscape of DDR mutations was described, as shown in Supplementary Fig. S5a. Twenty of the 65 patients had DDR pathogenic deficiency (*TP53* not included), mainly involving *NBN*, *PALB2*, *RAD50*, etc. However, no significant difference in PFS or OS was observed between patients with or without DDR mutations (Supplementary Fig. S5b).

#### dMMR, TMB, and PD-L1 expression

Only one patient in this study had dMMR, and this patient experienced complete resection (R0) and a best response of pathologic complete response (pCR), achieving a long survival exceeding 26.0 months (Supplementary Fig. S6). Among all included patients, NGS analysis showed that the TMB range was 0–70.9 mutations per megabase (Mb), and the median TMB value was 2.92 mut/Mb. The mean TMB values were 3.85 and 6.97 in the non-response and response groups, respectively, but this difference was not statistically significant (Fig. 2c). Moreover, the median PFS and OS were 5.0 months and 7.5 months in patients with TMB-H (≥ 2.92 mut/Mb), respectively. In patients with TMB-L (< 2.92 mut/Mb), the median PFS and OS were 6.3 months and 9.3 months, respectively. No significant correlation between TMB and the PFS or OS of patients was observed (Fig. 2c). Furthermore, PD-L1 status was determined in 56 cases using the 22C3 antibody. Overall, 24 (42.9%) patients had a PD-L1 CPS ≥ 1, and 16 patients (28.6%) had a CPS ≥ 5. We divided the patients into two groups according to PD-L1 expression level. The ORR of patients with higher PD-L1 expression (TPS ≥ 5% or CPS ≥ 5) reached 56.3%, while it was only 25.0% in the group with lower PD-L1 expression (TPS < 5% and CPS < 5) (*P* = 0.03). Consistently, the chi-square test showed a significant correlation between high PD-L1 expression and treatment response (CR or PR) (Fig. 2b). However, patients with higher PD-L1 expression did not achieve a significantly improved PFS or OS survival according to Kaplan–Meier analysis (Fig. 2b), and the association between PD-L1 expression and OS was identified by receiver operating characteristic (ROC) curve analysis (Supplementary Fig. S7). Interestingly, a PDAC patient with liver metastasis, who had PD-L1 expression with a TPS = 20% and CPS = 30, experienced a deep response and achieved long-term survival exceeding 19 months (Supplementary Fig. S4b).

#### Serum cytokines

To investigate the impact of serum cytokine levels on the prognosis of patients included in this study, we also detected baseline (pre-treatment) and post-treatment levels of a series of 17 serum cytokines. Except for interleukin-8 (IL-8), MCP-1 and MIP-1β, the detection rates of the other cytokines in the serum of these patients were rather low. We found no significant changes in the levels of IL-8, MCP-1 and MIP-1β from baseline to post-treatment (Fig. 2d). However, the baseline serum IL-8 level (*P* = 0.0035) and MCP-1 level (*P* = 0.035) in the response group was significantly lower than that in the non-response group (t-test), and a remarkably reduced baseline IL-8 level was observed in patients who achieved the median OS (≥ 8.9 months; Fig. 2d). In contrast, MCP-1 and MIP-1β showed no significant effect according to the subgroup on the treatment response or survival time (Fig. 2d). Notably, a higher baseline level of IL-8 (cutoff by the mean value) predicted a worse OS (Fig. 2d). These data suggest that the baseline serum IL-8 level could be a predictive biomarker for treatment response in pancreatic cancer patients undergoing chemotherapy combined with immunotherapy.

#### Tumor microenvironment analysis using CmTSA staining

(1) Identification and Comparison of Cell Components in TME Between Responders and Non-Responders.

**Fig. 3:**
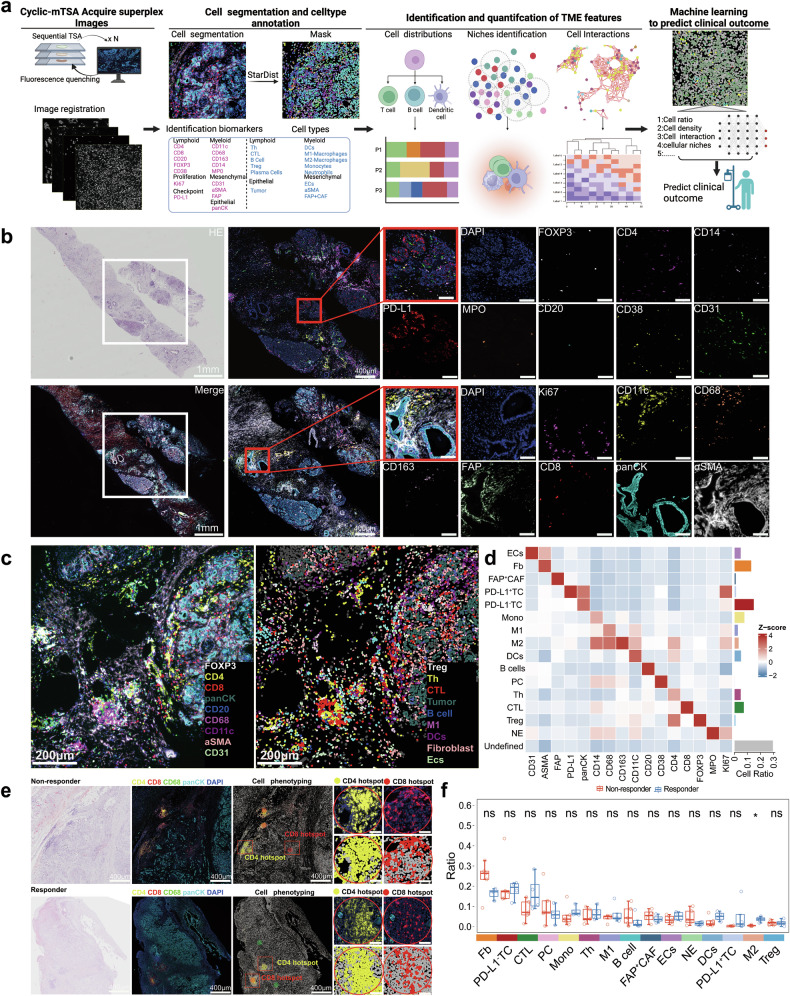
CmTSA staining workflow and comparison of cell components of TME between responders and non-responders. **a** Schematic of the cyclic multiplexed Tyramide Signal Amplification (CmTSA) workflow applied to TME analysis. Samples were subjected to sequential multiplex staining, and data were acquired through cyclic antibody stripping. Image registration was performed before cell segmentation and lineage assignment using tools like StarDist. Biomarkers were used to identify specific cell types within the TME, including lymphoid cells (e.g., CD3, CD8), myeloid cells (e.g., CD68, CD14), and stromal cells (e.g., FAP, αSMA). Spatial analysis of cell distributions, niches, and interactions was conducted, followed by integration into machine learning models to predict clinical responses. Figure 3a created with BioRender.com. **b** Representative multiplex imaging of protein expression alongside a corresponding haematoxylin and eosin (H&E) stain, demonstrating the expression of specific immune and stromal markers (right panels). Scale bars (left to right): 1 mm, 400 μm, 100 μm. **c** Multiplex immunofluorescence imaging (left) illustrates the spatial localization of immune and stromal markers. The artificial intelligence (AI)-identified mask (right) provides automated annotation of cell types within the TME. **d** Heatmap showing Z-score normalized mean intensities of immune markers across various identified cell subtypes, based on cell segmentation. The cell subtypes include CD20^+^ B cells (B), CD11c^+^ dendritic cells (DCs), CD31^+^ endothelial cells (ECs), FAP^+^ cancer-associated fibroblasts (FAP^+^ CAF), αSMA^+^ fibroblasts (Fb), CD68^+^ CD163^−^ macrophages (M1), CD38^+^ plasma cells (PC), CD14^+^ mature myeloid cells, MPO^+^ neutrophils, PD-L1^+^ tumor cells (PD-L1^+^ TC), PD-L1^−^ tumor cells (PD-L1^−^ TC), CD8^+^ cytotoxic T cells (CTL), CD4^+^ T helper cells (Th), CD4^+^ Foxp3^+^ regulatory T cells (Treg), Ki67^+^ proliferating cells, and other undefined cell types. **e** Comparison of cell phenotyping in CTL-rich regions (hotspots) between non-responders (top panels) and responders (bottom panels). The H&E staining (leftmost panels) provides an overview of the tissue structure. Fluorescent images display labeled markers for CD4 (red), CD8 (green), CD68 (yellow), panCK (cyan), and DAPI (blue). Scale bars (left to right): 400 μm, 100 μm. **f** Comparison of cell ratios of hot spots between responders (*n* = 4) and non-esponders (*n* = 5). Statistical significance is indicated as: ‘ns’ (not significant) and ‘*’ (*P* < 0.05). Data are presented as mean ± s.e.m

The TME plays a crucial role in tumor progression and response to treatment. It consists of diverse cell types, cell-cell interactions, and is organized into complex functional niches. To understand this complexity in our study, we utilized the superplex protein imaging-based cyclic multiplexed Tyramide Signal Amplification (CmTSA) analysis pipeline. Using the CmTSA technique, we profiled 17 proteins in formalin-fixed paraffin-embedded (FFPE) tissue sections from patients’ samples, collected as baseline pre-treatment samples (Fig. 3a). The analysis identified and segmented 16 cell types and their proliferating status (Ki67^+^), including PD-L1^+^ tumor cells (PD-L1^+^ TC), PD-L1^−^ tumor cells (PD-L1^−^ TC), lymphoid cells (T helper cells [Th], cytotoxic T lymphocytes [CTL], regulatory T cells [Treg], B cells, and plasma cells), myeloid cells (M1 macrophages [M1], M2 macrophages [M2], dendritic cells [DCs], neutrophils, and monocytes), mesenchymal cells (endothelial cells, fibroblasts, and FAP^+^ fibroblasts), and others (Fig. 3a–d).

We aimed to assess the relationship between the cellular composition and frequency of these immune-related cells and treatment response. However, the distribution of immune cells varied significantly across the TME. For instance, lymphoid cells and macrophages tended to cluster at specific areas, often at the invading edge. Therefore, selecting random regions of interest (ROIs) would not accurately represent the immune features of individual patients. Given the importance of CTL in anti-PD-L1 immunotherapy response, we specifically chose CTL-rich areas (hotspots) as ROIs for each patient (*n* = 1–3 ROIs per patient) (Fig. 3e). In our comparison of cell frequencies between typical responders (*n* = 4) and non-responders (*n* = 5), M2 macrophages were the only cell type that showed a difference (Fig. 3e, f). However, due to the low frequency of M2 macrophages across all samples (< 6%), their biological relevance remains uncertain and may not reliably indicate differences between groups (Fig. 3f). Nonetheless, M2 macrophages might play a critical role in the TME. Further higher order analysis including this cell type will be conducted to validate their potential significance.

(2) Identify and Quantify Multi-cellular Niches within the TME of Responders and Non-Responders.

**Fig. 4:**
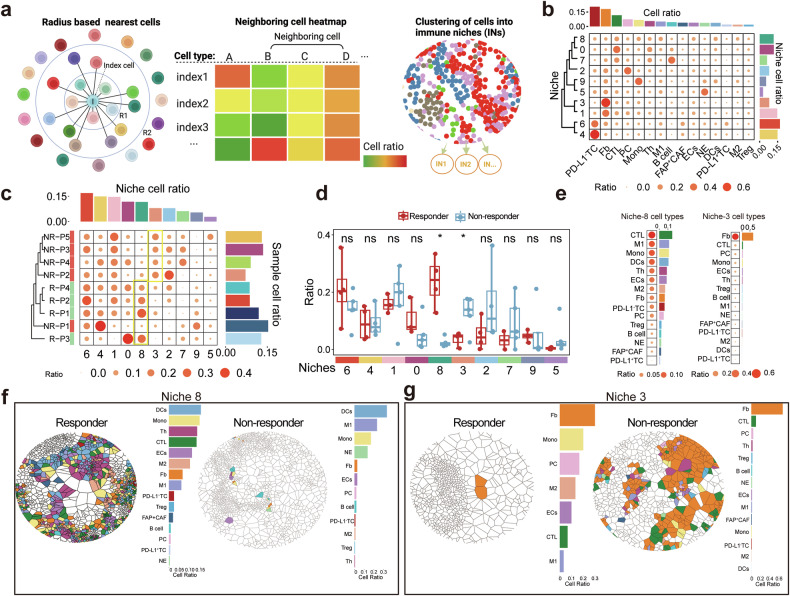
Identify and quantify multi-cellular niches within the TME of responders and non-responders. **a** Defination of Immune Niches (INs) in the tumor microenvironment (TME). Radius-based network depicting an index cell surrounded by neighboring cells within a fixed radius (left). Heatmap showing the ratio of neighboring cell types around each index cell (center). Cells are clustered into distinct INs using K-means clustering (right). Figure 4a created with BioRender.com. **b** The ratios of various cell types, such as CTL, B cells, macrophages, and fibroblasts, are compared across niches. The heatmap reveal the cell compositions of 10 distinct INs between responders (*n* = 4) and non-responders (*n* = 5). **c** The heatmap shows the distribution of niches (IN-0 to IN-9) in responders (R) and non-responders (NR). Unsupervised clustering reveals distinct niche compositions, with IN-3 enriched in non-responders and IN-8 more prevalent in responders. **d** Box plot comparing the ratios of niches (IN-0 to IN-10) between responders (red) and non-responders (blue). IN-8 is significantly higher in responders, while IN-3 is more prevalent in non-responders (*P* < 0.05). Data are represented as mean ± SEM. **e** Cell type composition of IN-3 and IN-8 niches. IN-8 is enriched with cytotoxic T lymphocytes (CTL), M1 macrophages, monocytes, and DCs, while IN-3 is dominated by fibroblasts, followed by CTL and plasma cells. **f** Spatial cell map of IN-8 comparing responders (left) and non-responders (right). **g** Spatial cell map of IN-3 comparing responders (left) and non-responders (right)

The various cell components within the TME interact through ligand-receptor binding (e.g., MHC-II/MHC-I to TCR, CD80/CD86 to CD28/CTLA-4, and PD-L1 to PD-1) and cytokine secretion (e.g., IL-12, IFN-γ, IL-10, and TGF-β). These interactions, both known and unknown, facilitate the formation of functional multi-cellular structures, or immune niches (INs), within the TME (Fig. 4a). Immune niches reveal immune activities and functions within patients’ TME, providing a potential strategy to correlate specific TME features with treatment response^21^. To explore this, we conducted spatial clustering analysis on samples from 9 patients (4 responders and 5 non-responders) using CmTSA pipeline (Fig. 4b, c). We identified a total of 10 INs from the TME of these patients, each characterized by unique cell compositions and ratios, indicating distinct immune activities. Basing on this differential proportion of each INs within the 9 patients, we then performed unsupervised clustering of the patients. Unlike patient clustering basing on the cell components within the TME, this approach effectively distinguished responders from non-responders (Fig. 4b, c). Notably, IN-3 and IN-8 emerged as the most significant niches differing between these two groups (Fig. 4d, e). IN-3, associated with non-responders, is characterized by a high proportion of spatially clustering fibroblasts (Fig. 4e, g). Based on previous studies in PDAC, a dense fibroblast layer within the TME acts as a significant barrier to treatment, impeding the penetration of chemotherapy drugs and creating an immunosuppressive environment^22^. This likely explains the role of IN-3 in contributing to drug resistance and reducing the effectiveness of anti-PD-1 immunotherapy. Conversely, IN-8 is closely associated with responders and is not dominated by a single cell type (Fig. 4e, f). Its primary components include CTL, M1 macrophages, DCs, and Th (Fig. 4e, f). The spatial clustering of these cells within IN-8 suggests a coordinated and active immune response. The presence of high proportion of IN-8 may be an effective predictor of a favorable therapeutic outcome.

(3) IN-8 Indicates a Robust and Coordinated Immune Response within the TME.

**Fig. 5:**
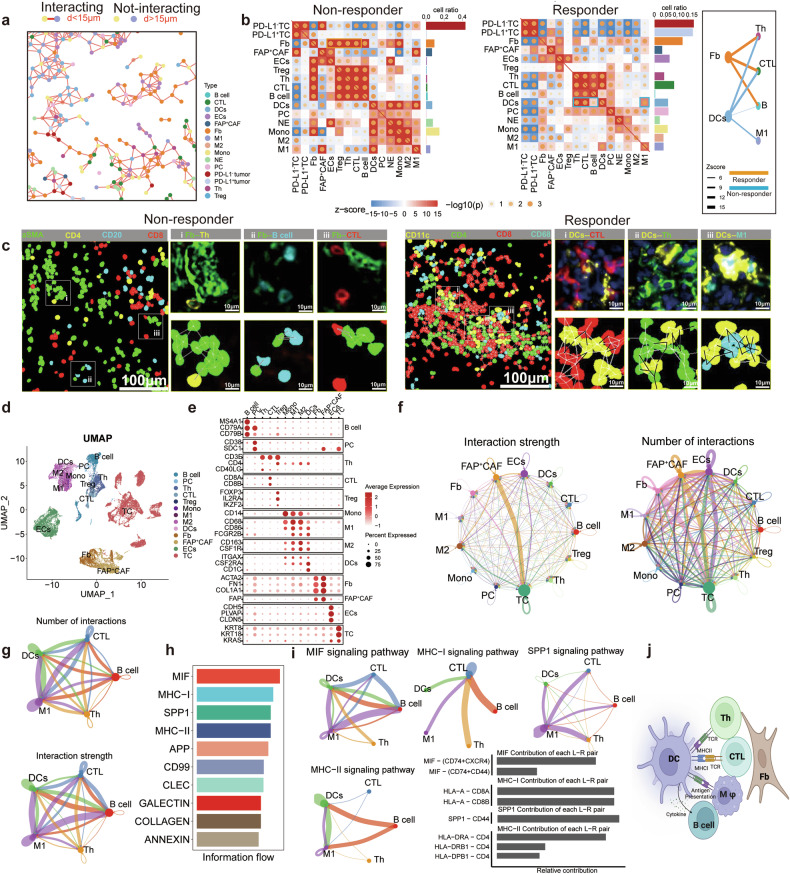
IN-8 indicates a robust and coordinated immune response within the TME. **a** Network diagram showing cell-cell interactions in the TME. Cells within <15 μm are connected, while cells farther than 15 μm apart are not connected. **b** Heatmaps of cell-cell interaction strengths: Responders (right) show DC interactions with CTL, Th, and M1 macrophages, while non-responders (left) show DC interactions with neutrophils, monocytes, and M1 macrophages, plus strong fibroblast-T cell interactions. **c** Illustrative depict the spatial interaction of Fb--Th (i)/ Fb--B (ii)/ Fb-CTL (iii) in non-responder (left), and DC-- CTL (i)/ DC--Th (ii)/ DC--M1 (iii) (right) in responder. **d** UMAP plot showing the clustering of 13 identified cell types from scRNA-seq analysis, including various immune cells (e.g., B cells, CTL, DCs), fibroblasts, endothelial cells, and tumor cells in the TME (*n* = 33085 cells across 24 patients). **e** Dot plot visualizing the expression of key marker genes across these cell populations. **f** Network diagrams comparing cell-cell interactions in the TME, illustrating interaction strength (left) and number of interactions (right) between various cell types. **g** Network diagrams illustrating the interaction strength (top) and the number of interactions (bottom) between key immune cells (DCs, CTL, Th, M1). **h** Ligand-receptor pairing analysis identified the top 10 pathways, highlighting the top contributors to immune cell communication, including MIF, MHC-I, SPP1, and MHC-II pathways. **i** Network diagrams show interactions in major pathways (MHC-I, MIF, SPP1) among immune cells like CTL, DCs, M1 macrophages, Th, and B cells. Bar graphs display the contribution of specific L-R pairs. **j** Schematic representation a coordinated anti-tumor response, orchestrated by immune cell interactions (DCs with Th, CTL, M1 macrophages, and B cells) in IN-8. Figure 5j created with BioRender.com

To understand the spatial arrangement and molecular communication among CTL, M1 macrophages, DCs, and Th within the IN-8, we constructed a spatial network linking all cells within a 15 μm distance, considering them as directly interacting (Fig. 5a). We quantified the intensity of these interactions by measuring the number of cell-cell connections within the TME of responders and non-responders. Notably, in responders, DCs frequently interacted with CTL, Th, and M1 macrophages, while in non-responders, DCs were more commonly associated with neutrophils, monocytes, and M1 macrophages (Fig. 5b, c). This suggests that, in the IN-8 of responders, DCs act as a key intermediary, facilitating interactions within the DC-CTL-Th-M1 cluster. This role is consistent with DCs’ function in antigen presentation, priming T-cell responses, and contributing to cancer control through adaptive immunity^23^. To further investigate the molecular interactions of these cells within PDAC, we utilized scRNA data from 24 PDAC patients, identifying 13 cell types, including DCs, CD8^+^ T cells (CTL), CD4^+^ T cells (Th), B cells, and macrophages, which are major components of IN-8 (Fig. 5d, e). Our analysis revealed that the most active cell-cell interactions occur between fibroblasts, (including other cancer-associated fibroblasts [CAFs]) with other cells^24^ (Fig. 5f). This is not surprising but rather consistent with the typical immunosuppression feature of PDAC^25^. To specifically examine the potential molecular interactions among DCs, CTL, Th, M1 macrophages, and B cells, we extracted and analyzed data pertaining to these cell types. The results indicated that, although less pronounced than interactions with fibroblasts, significant molecular interactions were present in the TME of PDAC patients (Fig. 5f, g). Further ligand-receptor pairing analysis identified MHC-I, MHC-II, MIF and SPP-1 as the top four pathways, suggesting robust antigen presentation and immune regulation (Fig. 5h, i). Overall, the presence of IN-8, characterized by the spatial clustering of CTL, M1 macrophages, DCs, B cells, and Th, along with the activation of MHC-I, MHC-II, and MIF reflects a robust and coordinated immune response (Fig. 5j). This suggests active antigen presentation and CTL-mediated tumor cell targeting, highlighting a potentially effective immune response. A high proportion of IN-8 within the TME suggests that the patient’s immune system is primed to respond to PD-L1-targeted therapies, making IN-8 a potential spatial predictor for immunotherapy response (Fig. 5j).

(4) DCs and Their Interactions with CTL and Th as Dominant Predictors of Treatment Response and Survival.

**Fig. 6:**
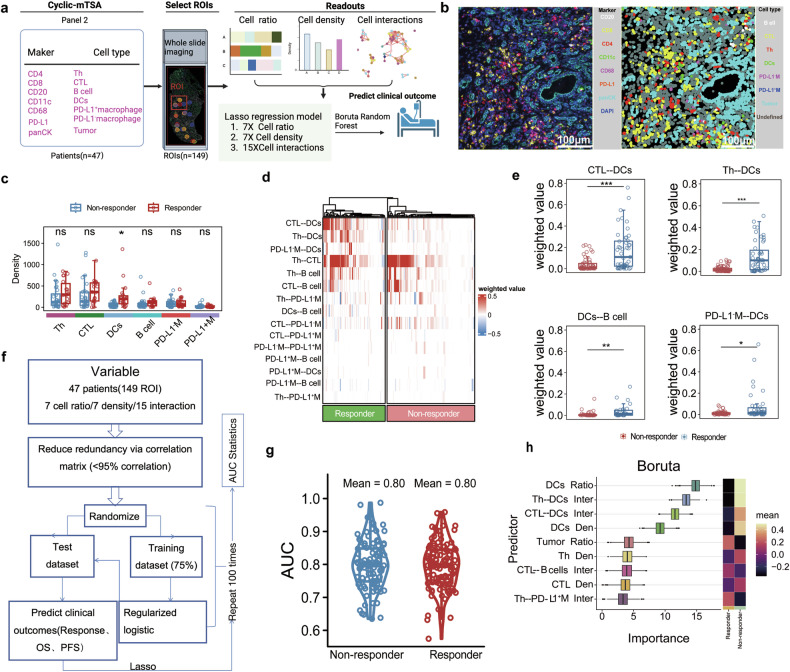
DCs and their interactions with CTL and Th as dominant predictors of treatment response. **a** Schematic representation of the CmTSA workflow on 47 patient samples and analytical strategy. Figure 6a created with BioRender.com. **b** Representative images showing multiplexed fluorescence staining of a clinical specimen (left): DAPI (nuclei), pan-cytokeratin (panCK^+^), cytotoxic T cells (CTL, CD8^+^), CD4^+^ effector T cells (Th, CD4^+^), B cells (CD20), PD-L1^+^ macrophage (PD-L1^+^ M), PD-L1^−^ macrophage (PD-L1^−^ M), dendritic cell (CD11c^+^). The MASK panel represents the segmentation and identification of different cell types within the tissue (right). **c** Quantitative analysis of cell density between responders and non-responders. Only dendritic cells (DCs) showed a significant difference (*P* < 0.05), while other cell types showed no significant variation (ns) among the response groups. Data are represented as mean ± SEM. **d** Heatmap showing cell-cell interaction intensities across responders and non-responders, based on 149 ROIs from 47 patients. Interactions between DCs and other immune cells, such as CTL, Th, and PD-L1^−^ M, are stronger in responders (green) compared to non-responders (red). **e** Higher interaction scores (CTL--DCs, Th--DCs, DCs--B cells, PD-L1^−^ M--DCs) correlate with better response outcomes. Comparison of cell-cell interaction intensities between responders (blue) and non-responders (red) for key cell pairs (CTL--DCs, Th--DCs, DCs--B cells, PD-L1^−^ M--DCs). The box plots show significantly higher interaction higher interaction scores in responders for CTL--DCs (*P* < 0.05), Th--DCs (*P* < 0.05), DCs--B cells (*P* < 0.05), and PD-L1^−^ macrophages (PD-L1^−^ M)--DCs (*P* < 0.05). Data are represented as mean ± SEM. **f** Analytical process for predictive modeling based on multi-layered multiplexed imaging data. **g** Violin plots illustrating the distribution of AUC values for the predictive models applied to responders and non-responders. **h** Boruta feature importance analysis identifying key predictors of treatent response (binomial p-value < 0.01), including cell ratios, cell densities (e.g., DCs Den, CTL Den), and cell-cell interactions (e.g., Th--DCs Inter, CTL--B cells Inter). Inter, interaction; Den,density

To further evaluate the potential of IN-8 as a predictor of response in advanced PDAC patients treated with combined chemotherapy and anti-PD-1 immunotherapy, we conducted a multi-parameter analysis on all patients enrolled in this clinical study (toripalimab + GnP). IN-8 is primarily composed of Th, CTL, B cells, DCs, macrophages, and tumor cells, clustered at varying ratios. To accurately represent the internal variables of IN-8, we performed CmTSA staining on available tissue samples (*n* = 47), using a customized panel of seven markers (CD4, CD8, CD20, CD11c, CD68, PD-L1, and panCK) to identify the specific cell types within IN-8. We measured and quantified three sets of internal variables: cell ratio, cell density, and cell-cell interactions (Fig. 6a, b). Among the seven identified cell types in the TME of all evaluated patients (ROI = 149, patients = 47), only the cell ratio of DCs were slightly higher in responders compared to non-responders (Fig. 6c). However, a significant difference was observed in the intensity of DCs interacting with Th, CTL, macrophages, B cells, and PD-L1^−^ macrophages, which was notably higher in responders (Fig. 6d, e). Next, we utilized a LASSO regression model to conduct a multi-parameter regression analysis, incorporating all quantified variables (7 cell ratios, 7 cell densities, and 15 cell-cell interactions) to assess the reliability of predicting patient responses. The data (149 ROIs) were split into training (75%) and test (25%) datasets, and regularized logistic regression models were applied (Fig. 6f). The analysis showed a similar mean area under the ROC curve (AUC) of 0.8 for both responders and non-responders, indicating that immune cells and their interactions significantly influence treatment response (Fig. 6g). Further analysis using the Boruta algorithm identified 12 major predictors contributing to overall model performance, with the DCs ratio and DCs interactions with CTL and Th being the most significant (Fig. 6h). Additionally, we analyzed the relationships between interactions of DC-Th-CTL and PFS as well as OS in PDAC patients (*n* = 47). In patients with longer OS (≥ 9.3 months) and longer PFS ( ≥ 6.1 months), higher cell-cell interaction intensities between key immune cells, including PD-L1^−^ macrophages (PD-L1^−^ M) and DCs, and Th with DCs (Supplementary Fig. S8a, e). Predictive models demonstrated high performance in distinguishing longer and shorter OS/PFS with mean AUC values of 0.77 for OS and 0.79 for PFS (Supplementary Fig. S8c, g). Boruta feature importance analysis identified DC-related interactions, such as CTL and PD-L1^−^ M interactions, as key predictors of survival outcomes (Supplementary Fig. S8d, h). This underscores the role of DCs as a dominant factor in predicting treatment response and survival outcomes in PDAC patients, highlighting their potential as a key driver of immune response and a valuable target for optimizing immunotherapy strategies.

## Disscussion

To our knowledge, this study is the first clinical study evaluating the addition of toripalimab to chemotherapy as a first-line treatment for newly diagnosed locally advanced and advanced PDAC. The results showed that toripalimab combined with GnP chemotherapy was safe and exhibited modest efficacy in these patients. The spectrum, frequency, and severity of TRAEs and irAEs were generally similar to those in previous reports^5,17^, and no unexpected TRAEs occurred. The most common TRAEs were associated with chemotherapy, and the grade 3/4 TRAEs were mainly hematologic and generally transient. No grade 4 irAEs were observed, and the reported irAEs were manageable with appropriate treatment. Thus, adding toripalimab to GnP chemotherapy showed a favorable safety profile in the present study.

Among the 72 PDAC patients in this study, the 12-month OS rate was 31.9% (23/72) and the median OS and PFS were 8.9 months and 5.6 months, respectively. These results were comparable to the AG group in the MPACT study^5^. Equally notable was that the proportions of patients with ECOG performance scores of 1–2 and with a greater metastatic tumor burden were relatively higher in our study compared with the MPACT study. Specifically, the efficacy results of the present study were consistent with those of several phase I/II single-armed studies that used an ICI plus chemotherapy to treat mPDAC. In the CCTG PA.7 phase II trial, the gemcitabine and nab-paclitaxel with durvalumab and tremelimumab group achieved a median OS of 9.8 months and nearly 12-month OS rate of 40%^18^. In a phase I study, PDAC patients treated with nivolumab combined with nab-paclitaxel plus gemcitabine had a median OS of 9.9 months and nearly 12-month OS rate of 30%^17^. Moreover, the randomized phase II PRINCE trial reported that the primary endpoint of 1-year OS was met by the group treated with nivolumab plus AG (*n* = 34, 1-year OS of 57.7% compared to historical control of 35%) and was not met by the group treated with sotigalimab plus nivolumab and AG (*n* = 35, 1-year OS of 41.3%)^26^. We observed an ORR of 33.3% (1 CR, 23 PR), indicating that the combination of gemcitabine, nab-paclitaxel and toripalimab did not result in significant improvement over the existing standard chemotherapy regimen. They suggested that these treatment regimens were not appropriate for all mPDAC patients, did not offer significant clinical benefit and that a biomarker-based selection strategy is needed^26^. While our study did not demonstrate a survival benefit relative to the previous MPACT study^5^, the addition of toripalimab to GnP did not increase toxicity and resulted in a higher ORR in patients with characteristic biomarkers. Therefore, these findings hold significant value for future clinical trial designs targeting selected populations. To our knowledge, this is the first prospective study investigating the addition of toripalimab to an established chemotherapy backbone, with an acceptable safety profile in PDAC. Notably, we employed CmTSA staining to analyze the TME, identifying potential predictive markers for favorable outcomes.

Consistent with the findings of previous reports^27–29^, we also observed a series of frequent mutations in PDAC, including in KRAS, TP53, SMAD4, etc. However, our examination of SNVs, DDR status, and TMB level did not identify any biomarkers that could predict treatment response and survival. A particular concern was that the overall TMB level of pancreatic cancer is low (from available data of 55 patients), and few patients in our study had a TMB >10 mut/Mb (3/55, 5.5%), with most patients having a TMB <5 mut/Mb (41/55, 74.5%). In a previous study with a larger sample size (*n* = 161), a very similar TMB level was detected in PDAC, with 7.5% of patients (12/161) having a TMB >10 mut/Mb^30^. At present, we consider that it is still difficult to evaluate the role of TMB in the response to immunotherapy plus chemotherapy due to the limited TMB-high population among PDAC patients. Consequently, at present, the National Comprehensive Cancer Network (NCCN) guidelines recommend that immunotherapy can be used for pancreatic cancer patients with MSI-H or TMB ≥10 mut/Mb, which is based on the results of the Keynote158 study^31^. Data from more cases are needed, but we expect that immunotherapy plus chemotherapy is a feasible scheme for PDAC with MSI-H. Additionally, PD-L1 expression is the most critical indicator for utilizing ICIs, and the PD-L1 expression score is applied in the prescription guide for anti-PD-1 treatment in non-small cell lung cancer, gastric cancer, bladder cancer, etc., but not known for PDAC^32^. In our study, the ORR among patients with higher PD-L1 expression was greater compared with that among patients with lower expression of PD-L1 (56.3% vs 25.0%, *P* = 0.03). However, adequate statistical power for OS or PFS analysis was lacking, probably due to the small sample size. In conjunction with the reported clinical studies, immunotherapy targeting PD-1/PD-L1 or CTLA-4 has demonstrated limited efficacy against pancreatic cancer if not selected using suitable biomarkers^17,18,26^, and shown potential only in a small subset of patients^33^. Additionally, we demonstrated that a lower baseline level of IL-8 predicted a better treatment response and longer survival. Recent studies also reported that elevated serum IL-8 correlates with reduced clinical benefit from ICIs^34,35^. The results indicate that the baseline serum IL-8 concentration could serve as a valuable biomarker for predicting treatment response to immunotherapy plus chemotherapy among patients with PDAC. Although 17 serum cytokines were tested, levels of key cytokines (IL-2, IL-12, IFN-γ, TNF-α, IL-10) were too low for analysis, likely due to patient characteristics or testing processes. Consequently, serum cytokines showed limited predictive value for patient outcomes. We further analyzed the correlation between serum cytokine profile and TME. On available data from 27 patients, we found that the density of Th was negatively correlated with IL-8 levels, and no correlation between TME and MCP-1 as well as MIP-1 (Supplementary Fig. S8i).

Notably, our exploratory investigation indicated this treatment could be beneficial for a subset with specific features. These potential biomarkers, including DC-Th-CTL enriched immune niche and their intimate spatial interactions in TME, high PD-L1 expression in tumor, were associated with response. Importantly, spatial interactions among various immune cells and tumor cells in TME, could effectively distinguish responders from non-responders. These findings in keeping with previous reports^36,37^, imply that immune cell subset activity harbored within the DCs-enriched niche could contribute to positive immunotherapy response on the one hand. Heterogeneous immunosuppression was a universal hallmark of the pancreatic TME^33^, which was also confirmed in our findings. Therefore, it is well established that existence of a baseline immune-promoting phenotypes and the activation of preexisting immunity determines the response to immunotherapy in these special PDAC patients. We speculate that spatial DC-Th-CTL interactions enrich for these tumor-reactive T cells. The predictive power of DC-Th-CTL interactions may therefore be explained by their close alignment with the underlying function of both the participant cells and the wider immune response. Immune cell cluster can also be observed in other solid tumors. Espinosa-Carrasco group demonstrated that CD4^+^ T cells must engage with CD8^+^ T cells and DCs, forming a three-cell-type cluster, be able to eliminate cancer cells^21^. CD8^+^ T cell effector differentiation and activation required CD4^+^ T cells, meanwhile, CD4^+^ T cells during the effector phase could license CD8^+^ T cell cytotoxicity and CD8^+^ T cell-mediated cancer cell elimination^21^. Naturally, the question of how dendritic cell (DC), Th and CTL communicate with each other to form this specific niche and subsequently regulate the following immune activity remains to be fully addressed. It was reported that some specific chemokines could enhance immunity by recruiting naive CD8^+^ T cells to sites of CD4^+^ T cell-dendritic cell interaction within antigen-draining lymph nodes, leading to optimal CD8^+^ T cell responses^38^. DC was activated by CD4^+^ T cells through CD40L-CD40 interactions, enhancing B7 and CD70 expression on DC; importantly, both CD4^+^ T cell and a CD8^+^ T cell recognized their respective antigens on the same DC^39^. DCs enhance CD4^+^ T-cell activation through antigen presentation, which increases CD40L expression, subsequently leading to CD40L-CD40 mediated boosting of DCs’ co-stimulatory signals CD86 and CD70, which interact with CD28 and CD27, respectively, on CD8^+^ T cells. This interaction, along with cytokines like IL-12 and IL-15, promotes the differentiation of CD8^+^ T cells into effector CTL^39^. Altogether, these findings reveal a crucial role for the unique DC-Th-CTL interactions and their spatial positioning within the TME, where CD4^+^ T cells endow CD8^+^ T cells with the ability to determine the effectiveness of PD-1 antibody-mediated anti-tumor immunity.

In contrast, we found that higher fibroblast-other immune cells (Treg, B cells, CTL and Th) interactions were observed in non-responders compared with responders, suggesting that the patients with fibroblasts enriched TME achieved less therapeutic benefit. It was well demonstrated that besides acting as a barrier to drug delivery and effector immune cell infiltration, cancer-associated fibroblast (CAF) could create dense desmoplastic stroma, thus forming an immunosuppressive TME in pancreatic cancer^22^. Therein, both the fibroinflammatory desmoplastic stroma promotion of T-cell exclusion and the highly heterogeneous CAF compartment contribute to the immunosuppressive TME, leading failure of ICI in PDAC^40–42^. Various studies have implicated the fibrotic components of PDAC to worse response/prognosis of patients and thoroughly analyzed the underlying contributions of various subtypes of fibrotic components. Such as, Ge et al. had fully clarified the characteristics of CAF subtypes in the TME, and demonstrated that α-SMA^+^ CAFs were correlated with the poor survival rates of pancreatic cancer patients^43^. And Wang et al. found metabolic state CAFs (meCAFs) as the most prominent subtype in PDAC^44^, furthermore, their groups had identified LA2G2A^+^ meCAFs in promoting tumor immune escape by impeding the antitumor immune function of CD8^+^ T cells in PDAC^45^. In general, these results are consistent with the findings of this study, IN-3 was characterized by a high proportion of fibroblasts, and the most active cell-cell interactions were observed between fibroblasts with other cells. It was suggested that fibroblasts suppressed CD8^+^ T cells and CD4^+^ T cells and fostered tumor immune suppression. Importantly, IN-3 is closely associated with non-responders. Therefore, CAF-immune cell interaction is another important contributer to tumor proliferation and invasion in these non-responders. Moreover, a patient with an abundance of myeloid-derived cells, such as monocytes or neutrophils, did not achieve a response, indicating that neutrophils in the TME could increase levels of immunosuppressive molecules, including PD-L1^46^. Furthermore, in both the longer OS and longer PFS groups, patients with higher interaction intensities between DCs, T cells (CTL, Th), and PD-L1^−^ M cells exhibited better survival outcomes. Additionally, our predictive models for both OS and PFS showed strong discriminatory power. Meanwhile, the feature importance analysis further identified these key interactions as the most critical predictors of patient outcomes, highlighting the importance of immune cell communication within the TME in determining survival trajectories.

This trial has several limitations. This study was a single-arm trial with a limited sample size and was conducted without a control group. The predictive value of the detected biomarkers needs to be further verified through future randomized controlled trials with a much larger sample size, including an additional chemotherapy control arm. The predictive value of serum cytokines as prognostic biomarkers of outcomes in our PDAC patients was limited. Moreover, development of an optimal antibody panel is required, and extensive validation across a much larger sample size is necessary.

In conclusion, toripalimab combined with GnP exhibited acceptable tolerability and differentiated efficacy in patients with locally advanced or metastatic PDAC. Our findings indicated that DC count, and its spatial organization collectively determined the immunotherapy effect, and spatial interactions of DC-Th-CTL could be used as potential predictors to efficacy of toripalimab plus chemotherapy in locally advanced or metastatic PDAC.

## Materials and methods

### Study design and endpoints

This prospective, single-center, open-label Phase Ib/IIa clinical trial included patients with inoperable, locally advanced or metastatic PDAC who had not previously received systemic therapy. The primary objective was to investigate the safety and OS of toripalimab in combination with the GnP regimen as first-line therapy in these patients. The main secondary endpoints were the ORR, DCR, PFS and DoR. And we aimed to find valuable predictive biomarkers via analysis of immune microenvironment. This study was conducted in strict accordance with the requirements of Good Clinical Practice and the Declaration of Helsinki. The study protocol was reviewed and approved by the Clinical Trial Ethics Committee of West China Hospital, Sichuan University (Ethics Approval Letter No.: 2019 Clinical Trial Review No. 41). The trial was registered as No. ChiCTR2000032293^47^. All patients provided written informed consent prior to enrollment, and the study protocol was available online^47^.

### Patients

For this single-arm phase Ib/IIa clinical trial, patients were enrolled from West China Hospital, Sichuan University between April 26, 2020 and September 8, 2022. The key inclusion criteria were as follows: age 18-80 years; Eastern Cooperative Oncology Group (ECOG) performance status score 0-2; histologically confirmed, unresectable locally advanced or metastatic PDAC not previously treatment with systemic therapy. The trial protocol listing the full eligibility criteria is provided at 10.1186/s12885-020-07126-3 and provided in the Supplementary protocol and methods.

### Procedures

All enrolled patients received combination therapy with intravenous toripalimab 240 mg on day 1, intravenous nab-paclitaxel 125 mg/m^2^ on days 1 and 8, and intravenous gemcitabine 1000 mg/m^2^ on days 1 and 8, in a 21-day cycle. Treatment was stopped upon the occurrence of PD confirmed according to the Response Evaluation Criteria in Solid Tumors version 1.1 (RECISTv1.1) criteria, the occurrence of unacceptable adverse events, the presence of concomitant diseases that prevented continuation of treatment, investigator decision to withdraw the patient from the trial, patient withdrawal of informed consent, patient pregnancy, or patient noncompliance with the trial treatment or procedures (Supplementary Fig. S1a, S1b). After six cycles of combination therapy, therapeutic options (including toripalimab as maintenance treatment or continuous treatment) exist, depending on the choice of the clinician. Imaging evaluation, adverse events, dose reduction methods were provided in Supplementary Table S4, Supplementary protocol and methods and Supplementary Fig. S1b.

### Biomarker exploration

Baseline tumor tissue specimens, serum samples were obtained for biomarker analysis. DNA sequencing, PD-L1 expression, CmTSA analysis, and serum cytokine were detected as baseline biomarker. Serum cytokine at post-treatment was also detected. All detailed biomarker data testing and analysis process, including DNA sequencing, PD-L1 expression, cytokine detection and CmTSA analysis process, were available in the Supplementary protocol and methods.

### Statistical analysis

Sample size estimation was performed according to the Simon two-phase method, selecting error rates alpha equal to 0.05 (both sides) and beta equal to 20%. Given the 1-year OS rate (35%) observed in the MPACT study, success was defined as a 1-year OS rate of at least 55%. The plan included enrolling a total of 17 eligible patients in the phase I study, and if the 1-year OS rate was less than 6/17, further recruitment would be terminated. If the 1-year OS rate was more than 6/17 though, the trial would continue with enrollment of 32 patients in the phase II study. With a total estimated enrollment of at least 49 patients, allowing for withdrawal or loss to follow-up of 10% of patients, the number of patients that needed to be initially enrolled was 54.

The Kaplan–Meier method was utilized to analyze time-to-event variables (median OS, PFS, and DoR), with post-hoc analyses for calculating 1-year OS rates. The chi-square test and Fisher’s exact test were used to compare rates between groups. For numeric variables, Student’s t-test was used to identify differences between the two groups. All tests were two-sided, and P values <0.05 were considered statistically significant. Cox proportional hazards regression analysis was used to investigate association between biomarkers and OS, PFS, and baseline patient characteristics. Baseline data were collected using Microsoft Office Excel 2010. SPSS (version 26.0) statistical software and computing software R v4.0.2 were used for statistical analysis and graphics of the data.

## Supplementary information

Supplementary Materials

## Data Availability

The original data are available from the corresponding author upon request sharing by sending requests to the corresponding author Dan Cao (caodan@scu.edu.cn). Due to the patient privacy, clinical data were not publicly available, but could be accessed from the corresponding author. The remaining data are available in the Supplemental Materials. All computer codes utilized for analyzing spatial distributions, along with any further explanations, can be obtained from the corresponding author upon request.
