# Supplementary material for: Spatial interactions of immune cells as potential predictors to efficacy of toripalimab plus chemotherapy in locally advanced or metastatic pancreatic ductal adenocarcinoma: a phase Ib/II trial
Source: Signal Transduct Target Ther. 2024 Nov 25;9:321. doi: 10.1038/s41392-024-02031-8 (PMC11586424; doi:10.1038/s41392-024-02031-8)
Supplement: Supplementary file 1 — Supplementary Materials [file 41392_2024_2031_MOESM1_ESM.docx]

Supplementary Materials for

Spatial interactions of immune cells as potential predictors to efficacy of toripalimab plus chemotherapy in locally advanced or metastatic pancreatic ductal adenocarcinoma: a phase Ib/II trial

Ke Cheng^#^, Xiaoying Li^#^, Wanrui Lv^#^, Gang Zhao, Ruihan Zhou, Chen Chang, Heqi Yang, Ruizhen Li, Zhiping Li, Ye Chen, Cheng Yi, Ouying Yan, Chaoxin Xiao, Yi Zhang, Junjie Xiong, Zixin Huang, Weikang Shao, Xin You, Wenhao Guo, Du He, Wenwu Ling, Rui Wang, Bole Tian^*^, Chengjian Zhao^*^, Dan Cao^*^

Correspondence to: caodan@scu.edu.cn; chjianzhao@scu.edu.cn; tianbole@scu.edu.cn

**This PDF file includes:**

Protocol and methods

Figures. S1 to S8

Tables S1 to S4

Protocol and Methods

The full inclusion and exclusion criteria

The key inclusion criteria were as follows: age 18-80 years; Eastern Cooperative Oncology Group (ECOG) performance status score 0-2; histologically confirmed, unresectable locally advanced or advanced PDAC not previously treatment with systemic therapy; pancreatic cancer judged to be unresectable by imaging physicians and/or surgeons according to the criteria for unresectable pancreatic cancer in the eighth edition of the American Joint Committee on Cancer TNM staging system; in cases with postoperative recurrence and metastasis, the time from recurrence and metastasis to the end of postoperative chemotherapy was more than 6 months; adequate organ function including cardiac, renal and hepatic function; and no history of active autoimmune disease. The key exclusion criteria were as follows: severe autoimmune disease/immunodeficiency and participation in another clinical trial within 6 weeks before enrollment.

Procedures

Imaging evaluation by computed tomography (CT) or magnetic resonance imaging (MRI) was performed every two cycles. Efficacy was evaluated according to RECIST 1.1 criteria by two independent radiologists. Adverse events were graded according to the National Cancer Institute Common Terminology Criteria for Adverse Events (CTCAE) Version 5.0. Gemcitabine and albumin-paclitaxel doses could be adjusted according to adverse reactions. The specific dose reduction methods are described in the study protocol, provided in **Supplementary Table S4**.

DNA extraction and sequencing

Next-generation sequencing (NGS) of DNA from tumor samples was performed using the 769 cancers gene-targeted panel (Genecast Biotechnology Co., Ltd., Wuxi, China). Formalin-fixed paraffin-embedded (FFPE) tissues were collected from each patient’s primary tumor or metastatic lesion, and matched whole blood DNA was collected for analysis. DNA was isolated from FFPE tissues using the black PREP DNA kit (Analytik Jena, Germany). Whole blood centrifugation (1600 × g) was performed for 10 min at room temperature for separation of lymphocytes. DNA was extracted from peripheral blood lymphocytes using the Tiangen Whole Blood DNA Kit (Tiangen, Beijing, China) according to the manufacturer’s instructions. Genomic DNA was fragmented into 150–200-bp fragments with a Covaris M220 Focused-Ultrasonicator (Covaris, USA). Fragmented DNA libraries were constructed using the KAPA HTP library preparation kit from the Illumina platform (KAPA Biosystems, USA) according to the manufacturer’s instructions. DNA libraries were captured with a designed 2M panel of the IDT library (Integrated DNA Technologies, USA), which included major tumor-related genes. The captured samples were then subjected Novaseq 6000 processing for paired-end sequencing.

Tumor mutational burden (TMB), microsatellite instability (MSI) and PD-L1 analysis

TMB is defined as the total number of somatic nonsynonymous mutations per million bases (Mb) in the coding region of the tumor genome, including point mutations and gene insertions or deletions. We filtered all samples for single-nucleotide variants (SNVs) according to the following rules: (i) non-splicing or exonic; (ii) depth <100× and allele frequency <0.05; (iii) allele frequency ≥0.002 in the Exome Aggregation Consortum (ExAC) database and genomad; and (iv) strand biased mutations in reads and other rules. Patients with well-established TMB testing were divided into two categories by median TMB value: TMB-high (≥ median value of TMB) and TMB-low (˂ median value of TMB). Seventy microsatellite loci of interest were examined and compared with genomic data from healthy individuals in a Chinese database. The number of microsatellite loci altered by somatic insertions or deletions was counted for each patient sample. MSI status was MSI-high (MSI-H) if the destabilizing site/passing site ratio was ≥0.3 or MSI-low (MSI-L)/ microsatellite stable (MSS) if the ratio was <0.3. Using the bam file obtained from mapping, reads containing exclusively short tandem repeat motifs were obtained, and then the allele length of each gold standard locus was calculated. MSI-H was considered when the number of unstable loci was ≥2, and MSI-L/MSS was considered when the number of stable loci was ≥4.

Tissue sections were first deparaffinized and rehydrated, followed by antigen retrieval using the specified retrieval buffer. The slides were then incubated with the primary PD-L1 antibody from the Dako PD-L1 IHC 22C3 pharmDx kit. After applying an HRP-conjugated secondary antibody, DAB chromogen was used for visualization. Finally, the slides were counterstained with hematoxylin, dehydrated, and mounted for evaluation. PD-L1 expression was analyzed using the FDA-approved 22C3 assay (Dako PD-L1 IHC 22C3 pharmDx). PD-L1 expression was determined by a pathologist and represented using the tumor-proportion score (TPS) or the combined positive score (CPS). PD-L1 TPS≥1% or CPS≥1 was considered PD-L1 positive. PD-L1 expression was defined as not available (NA) for patients whose collected tumor tissues could be evaluated for PD-L1 expression or for whom tumor tissues could not be collected.

Cytokine detection

Patient serum samples were collected during hospitalization (n=47), and liquid bead suspension chip analysis was performed (Bio-Plex Pro Human Cytokine Grp I Panel 17-Plex, M5000031YV) by Shanghai Huaying Biomedical Technology Co., Ltd. (China) using a Luminex 200 system (Luminex Corporation, USA) following the manufacturer’s instructions. The 17 cytokine screening panel includes: tumor necrosis factor alpha (TNF-α), interferon-gamma (IFN-γ), granulocyte colony-stimulating factor (G-CSF), granulocyte-macrophage colony-stimulating factor (GM-CSF), interleukin (IL)-13, IL-1β, IL-17, IL-2, IL-4, IL-5, IL-6, IL-7, IL-10, IL-8, IL-12, monocyte chemoattractant protein-1 (MCP-1), and macrophage inflammatory protein-1 beta (MIP-1β). As performed in previous reported studies^1-4^, the detection process was carried out following the Bio-Plex Pro™ Human Cytokine instructions using a BioPlex 200 machine (Bio-Rad) and the manufacturer’s protocol.

The detailed assay workflow was following:

Step 1 (sample preparation): serum samples were centrifuged at 10,000 rpm for 10 minutes, and the supernatant was diluted 4-fold with Sample Diluent. For analysis, 50 μL of each diluted sample was added to the assay plate. Step 2 (standard and control preparation): standards were reconstituted with 250 μL of sample diluent, vortexed briefly, and incubated on ice for 30 minutes. An 8-point standard curve (S1-S8) was prepared through serial dilution. Step 3 (bead-based assay setup): a Bio-Plex Pro Human Cytokine 17-Plex panel was used, targeting cytokines like TNF-α, IFN-γ, G-CSF, GM-CSF, various interleukins, MCP-1, and MIP-1β. Beads were rehydrated, diluted in Assay Buffer, and 50 μL was added to each well, followed by washing 3 times using a magnetic plate washer. Step 4 (incubation): 50 μL of standards, samples, or blanks were added to the wells, covered, and incubated on a plate shaker at 850 rpm, protected from light, for 30 minutes at room temperature. Step 5 (detection antibody and signal development): wells were washed 3 times, followed by the addition of 25 μL of diluted detection antibody to each well, incubating again at 850 rpm for 30 minutes. After another 3 washes, 50 μL of Streptavidin-PE was added and incubated for 10 minutes in the dark. Step 6 (data acquisition): after a final wash, 125 μL of assay buffer was added, and the plate was read using the Luminex 200 system, measuring fluorescence intensity for each cytokine. Step 7 (data analysis): the Luminex system software processed the fluorescence data, calculating cytokine concentrations based on the standard curve, and results were exported for further analysis.

Cyclic multiplexed tissue staining (CmTSA) and analysis strategy

(1) Cohorts and clinical specimens

﻿FFPE PDAC tissues were collected from the department of pathology, West China Hospital, Sichuan University. ﻿A total of 47 patients with available tissue samples in clinical trials. All participants or their legal guardians provided informed consent. Responders were defined as patients with a RECIST complete response (CR) or partial response (PR), while non-responders were defined as those with progressive disease (PD) or stable disease (SD).

(2) ﻿Deparaffinization and antigen retrieval

Biopsy or resection specimens of formalin-fixed and paraffin-embedded (FFPE) tissue samples, which were cut into 4 μm sections. In preparation for HE and CmTSA, the tissue sections were subjected to deparaffinization with xylene and subsequent rehydration through a series of graded ethanol solutions (﻿95%, ﻿85%, 75%, each for 3 minutes). The sections were initially placed in a staining jar containing purified water and rinsed three times, each for 3 minutes. Subsequently, the slides were subjected to a preheated water bath at 95°C, and antigen retrieval was facilitated through a 30-minute exposure to EDTA target retrieval solution pH 9.0 (ZLI-9068). Finally, they were washed three times with PBS, with each wash lasting 2 minutes.

﻿(3) Hematoxylin and eosin (H&E) staining

After deparaffinization and antigen retrieval, tissue sections are stained with hematoxylin to color the cell nuclei blue. A bluing step with a weak alkaline solution follows, enhancing the hematoxylin stain. The sections are then stained with eosin for pink to red contrast in the cytoplasm. After staining, sections are dehydrated through graded ethanol solutions, cleared in xylene, and mounted with a coverslip using a permanent mounting medium. This process results in blue-stained nuclei and pink-stained cytoplasmic components. Hematoxylin and eosin (HE) staining was performed on each sample to determine the presence of tumor by pathologists.

(4) Tissue processing for CmTSA staining

4.1 ﻿Background fluorescence quenching

Following deparaffinization and antigen retrieval, tissue sections were placed in the LUMINIRIS Fluorescence Quenching System (#MH030101). They were then treated with IRISKit^®^ HyperView quench buffer (#MH010301) and subjected to 600W fluorescence quenching for 8 minutes to minimize background fluorescence. After quenching, the sections were rinsed with PBS.

4.2 CmTSA staining

A hydrophobic barrier was drawn around the tissue sections using an immunohistochemistry pen. Antigen labeling was performed using the IRISKit^®^ HyperView multiplex immunostaining kit (#MH010101) with sequential application of the primary antibody (20 minutes), the primary antibodies used included PD-L1 (HUABIO; HA721176; 1:1000), CD3 (HUABIO; HA720082; 1:1000), CD68 (HUABIO; HA601115; 1:3000), CD8 (Immunoway; ABT304; 1:200) CD20 (HUABIO; HA721138; 1:1000), pan Cytokeratin (HUABIO; HA601138; 1:3000), CD4 (HUABIO; ET1609-52; 1:1000), CD11c (HUABIO; ET1606-19; 1:1000), FOXP3 (Abcam; ab20034; 1:800), MPO (Abcam; ab208670; 1:1000), CD31 (HUABIO; M15118; 1:2000), CD14 (HUABIO, ET161085, 1:1000), CD163 (HUABIO, ET1704_43, 1:2000), aSMA (HUABIO, ET1607_53, 1:5000), FAP (R381838, 1:1000), CD23 (HUABIO, HA721139, 1:1000), Ki67 (HUABIO; HA721115; 1:3000). Following that, the slides underwent a 20-minute incubation at room temperature with secondary antibodies (HRP Conjugated Goat anti-Rabbit IgG: HA1119; HRP Conjugated Goat anti-Mouse: IgG HA1120) and fluorescent dye (5–10 minutes per antibody). After each labeling, antibody stripping was done using the IRISKit^®^ HyperView Advanced Ab Stripping Kit. Tyramide-based fluorescent dyes used included DAPI (440–680 nm), FITC (520–680 nm), Cy3 (570–690 nm), and Cy5 (670–720 nm). Following the three antibody labels, DAPI was applied for nuclear staining. Sections were washed twice with PBS (3 minutes each) between reagents, mounted with a mounting medium, and imaged using the EVIDENT VS200 microscope at 20x magnification (resolution: 0.345 μm/px). The reagents and techniques employed in whole mount immunostaining are sourced from the IRISKit® HyperView multiplex immunostaining kit (#MH010101).

(5) Histological image acquisition

After imaging, quench the specific staining fluorescence while preserving DAPI for image registration. The quenching procedure is performed as previously described. Once quenching is complete, proceed with the next round of staining using three antibodies. A total of 47 histologic specimens were collected, sectioned, and stained. Slides were imaged at 20× magnification using SLIDEVIEW VS200 (OLYMPUS) by multiple participants who were blinded to the data analysis. After acquiring images from multiple rounds, an algorithm automatically identifies feature points in the DAPI channel. A rigid transformation (translation, rotation) is applied to achieve optimal overlap, minimizing errors. Images are then standardized to an 8-bit grayscale format (0 to 255), enabling proper display for subsequent registration, analysis, and further operations. Image registration by using Fiji ^5^.

(6) CmTSA staining images data analysis

6.1 Cell segementation

The CmTSA staining images were analyzed using QuPath ^6^. The QuPath software utilized unmixed images to learn the phenotyping algorithm (Stardist). The StarDist model was used for accurate cell nuclei segmentation in microscopy images. The pretrained StarDist model was imported into QuPath, and the preprocessed DAPI channel image was segmented using the model’s star-convex polygon approach. Mean fluorescence intensity for each channel was measured across nuclei, cytoplasm, membrane, and whole cell, enabling marker positivity filtering. The x and y coordinates of each cell's nuclear centroid were also extracted for calculating spatial relationships between cells.

6.2 Marker Thresholds for Cell Type Classification

For each multiplex image of FFPE sections, individual thresholds were set for each marker channel. The appropriate cellular location (e.g., nucleus or membrane) was selected, and a threshold within the 0–255 range was applied, classifying cells as marker-positive or marker-negative. This process was manually supervised, with marker-positive cells displayed using a predefined mask color. Given that some markers are shared among cell types and others require multiple markers for identification, an annotation rule was established for consistent, automated cell subtype classification. Cellular phenotypes were assigned based on typical lineage markers, population abundance, and staining quality^7^.

6.3 Regions of interest

We selected hotspot regions of T cells as regions of interest (ROIs) by whole-slide image observation. Responders (n=4) and non-responders (n=5) were stained with panel 1, each ROI covered approximately 4 mm², or a smaller area if the analyzable cancerous region was less than 4 mm², and the cell density in each region of interest (ROI) was calculated by normalizing the total cell counts by the total area (cells/mm²). 47 patients were stained with panel 2 and were analyzed with QuPath for tissue component segmentation and cell phenotyping. For each patient, we calculated the cell density in the tumor core by considering all ROIs from each respective area for the patient.

(7) Data Conversion and Standardization

All analyses utilized raw CmTSA staining images. Panel 1 Cells were classified into one of 15 distinct categories based on our selected markers (Fig.3): CD20^+^ B cells (B), CD11c^+^ cells (DCs), CD31^+^ cells (ECs), FAP^+^ fibroblast (FAP^+^ CAF), aSMA^+^ fibroblast (Fb), CD68^+^ CD163^−^ macrophages (M1), CD38^+^ plasma-cells (PC), CD14+ cells (Mature myeloid-cells), MPO (Neutrophil), PD-L1^+^ tumor cell (PD-L1^+^ TC), PD-L1-Tumor cell (PD-L1^−^ TC), CD8^+^ T cells (CTL), CD4^+^ T cells (Th), CD4^+^ T^+^ Foxp3^+^ (Treg), Ki67 and (undefined). Single-cell marker expressions were summarized using the mean pixel values for each channel (**Fig. 3**).

Panel 2 Cells were classified into one of 15 distinct categories based on our selected markers: cancer cells expressing pan-cytokeratin (panCK^+^), cytotoxic T cells (CTL, CD8^+^), CD4^+^ effector T cells (Th, CD4^+^), B cells (CD20), PD-L1^+^ macrophage (PD-L1^+^ CD68^+^), PD-L1^−^ macrophage (PD-L1^−^ CD68^+^), dendritic cell (CD11c^+^) (**Fig. 6**). All phenotyping and the subsequent quantification processes were conducted without knowledge of the sample identities or clinical outcomes. Data presented were not modified. For heatmap visualization, expression data were normalized to the 95th percentile, and z-scored cluster means were plotted.

(8) Cell interaction analysis

8.1 Single-cell RNA sequencing data

To investigate the interactions between various cell types. We re-analyzed the single-cell RNA-seq data from the study by Junya Peng et al ^8^, which included samples from 24 PDAC patients. In our analysis, we further refined the classification of various immune cells and cancer-associated fibroblast.

After normalization and scaling**，**the data were further reduced using Uniform Manifold Approximation and Projection (UMAP), allowing clear identification of various cell clusters, such as dendritic cells (DCs), T cells (Th, CTL), macrophages (M1, M2), B cells, and fibroblasts. Cluster markers were identified using differential ﻿FindAllMarkers function in ﻿Seurat package, which identified genes significantly enriched in each cluster^9^. Cell types were annotated based on the expression of known marker genes, verified by reference to established cell markers from existing literature^8,10^.

Cell-cell communication networks were analyzed using CellChat, which predict ligand-receptor interactions based on the expression profiles of each cell cluster ^9^. Interaction strength and the number of interactions between cell types were quantified and visualized using network diagrams, allowing identification of key cellular players in the immune response. The strength and extent of these interactions were further investigated, focusing on specific immune cell types such as DCs, CTL, Th, and macrophages. Information flow within these networks was assessed using key signaling molecules and pathways, identified by their contributions to cell-cell communication, such as MIF, MHC-I, SPP1, and MHC-II. Detailed signaling pathway networks were analyzed to understand how these pathways facilitate interactions.

The scRNAseq data analysis was performed using Seurat (version 4.0.1) in R (v4.3.1). The data were filtered to include cells that expressed a minimum of 100 genes and had no more than 25% of their reads mapped to mitochondrial genes.

8.2 Spatial network and cell interaction construction

To construct a spatial network of cells for analysis, we initially used the 'nn2' function from the RANN package (v2.6.1) in R (v4.3.1). Parameters were set with k=15 and a search radius of 15μm, considering the first 15 neighboring cells within this radius while excluding the focal cell ^7,11,12^.

The spatial architecture of cells within tissues is critical for understanding how individual cells organize into functional units. By analyzing the spatial locations of the relevant markers obtained by CmTSA, SOAPy (Spatial Omics Analysis in Python) ^13^ employs a comprehensive approach to analyze cell interactions by constructing a cell/spot network based on spatial locations and implementing two main scenarios: spatial proximity and spatial composition analyses. This preliminary network provided an initial visualization of spatial relationships between cells. For more detailed spatial analysis, we applied the Spatial Network Construction method from the SOAPy package, enabling a finer-scale examination of cell-type interactions and uncovering more complex biological phenomena. All analyses and visualizations were performed using Python (v3.9) and R (v4.3.1).

(9) Niche Definition and Niche Clustering

Identifying and characterizing cellular niches is essential for understanding the functional organization of tissues and how different cell types interact within their microenvironment. SOAPy is used to generate niches and subsequent niches analysis ^7,13^.SOAPy provides a robust framework for niche identification by integrating spatial composition analysis with tensor decomposition methods. Each niche is represented by the proportion of neighboring cell types, termed I-niche. Firstly**,** construct a network where each node is a cell, and edges represent spatial proximity. Secondly calculate the proportion of each cell type in the neighborhood of every cell to define its I-niches.

To group similar niches into clusters (C-niches) to identify common microenvironment patterns. Use clustering algorithms to categorize I-niches into distinct C-niches. Firstly, apply K-means or similar clustering algorithms to group I-niches based on their cell type proportions. Secondly, analyze the composition and distribution of C-niches across the tissue samples. Apply tensor decomposition methods to analyze high-order spatial data (e.g., Niche-Cell Type-Sample tensor). By integrating spatial proximity and composition analyses, SOAPy provides a comprehensive framework for dissecting the spatial organization of tissues, identifying key cellular interactions, and uncovering the intricate microenvironmental niches that underpin tissue function and pathology.

The detailed implementation steps can be found in our team's preprint “Pipeline for Assessing Tumor Immune Status Using Superplex Immunostaining and Spatial Immune Interaction Analysis”^7^. All analyses and visualizations were performed using Python (v3.9) and R (v4.3.1).

(10) Multivariate Modelling and Feature Selection

The multivariate modelling and variable importance were adapted from the method developed by ﻿Xiao Qian Wang et al ^12^. We utilized regularized logistic regression models (using the R package glmnet) to assess the predictive performance of aggregated tissue features for treatment response. Three sets of features were derived for each tumor, separating epithelial and TME compartments: cell phenotype densities, cell interaction metrics, and cell type ratio. From this, 29 variables were derived for single timepoints, retaining only variables with more than six unique values.

To reduce the feature space, we identified and grouped highly correlated variables (Spearman rank correlation >0.95) and selected one representative variable per group. Data were split into training (75%) and test (25%) sets, with regularized logistic regression models fitted to the training set and predictions made on the test set. The process was repeated 100 times to estimate AUC precision and derive 95% confidence intervals. Model performance on the blinded testing dataset was evaluated using AUC ranges.

For feature importance, we used the Boruta algorithm, which compared true values with randomly shuffled features to identify significant predictors^12^. This process, repeated 1,000 times, helped generate a final set of important variables based on a threshold of *P* <0.01, with importance values plotted to rank their significance.

**Reference:**

1 Rouanne, M. *et al.* BCG therapy downregulates HLA-I on malignant cells to subvert antitumor immune responses in bladder cancer. *J Clin Invest*. **132**, e145666 (2022).

2 Gerber, D. E. *et al.* Concentration-dependent Early Antivascular and Antitumor Effects of Itraconazole in Non-Small Cell Lung Cancer. *Clin Cancer Res*. **26**, 6017-6027, (2020).

3 Liu, W. *et al.* Cisplatin-stimulated macrophages promote ovarian cancer migration via the CCL20-CCR6 axis. *Cancer Lett*. **472**, 59-69, (2020).

4 Andaluz-Ojeda, D. *et al.* A combined score of pro- and anti-inflammatory interleukins improves mortality prediction in severe sepsis. *Cytokine*. **57**, 332-336, (2012).

5 Schindelin, J. *et al.* Fiji: an open-source platform for biological-image analysis. *Nat Methods*. **9**, 676-682, (2012).

6 Bankhead, P. *et al.* QuPath: Open source software for digital pathology image analysis. *Sci Rep*. **7**, 16878, (2017).

7 Xiao, C. *et al.* Pipeline for Assessing Tumor Immune Status Using Superplex Immunostaining and Spatial Immune Interaction Analysis. Preprint at https://www.biorxiv.org/content/10.1101/2024.08.23.609368v1 (2024).

8 Peng, J. *et al.* Single-cell RNA-seq highlights intra-tumoral heterogeneity and malignant progression in pancreatic ductal adenocarcinoma. *Cell Res*. **29**, 725-738, (2019).

9 Jin, S. *et al.* Inference and analysis of cell-cell communication using CellChat. *Nat Commun*. **12**, 1088, (2021).

10 Hutton, C. *et al.* Single-cell analysis defines a pancreatic fibroblast lineage that supports anti-tumor immunity. *Cancer Cell*. **39**, 1227-1244.e1220, (2021).

11 Geri, J. B. *et al.* Microenvironment mapping via Dexter energy transfer on immune cells. *Science*. **367**, 1091-1097, (2020).

12 Wang, X. Q. *et al.* Spatial predictors of immunotherapy response in triple-negative breast cancer. *Nature*. **621**, 868-876, (2023).

13 Wang, H. *et al.* SOAPy: a Python package to dissect spatial architecture, dynamics and communication. Preprint at https://www.biorxiv.org/content/10.1101/2023.12.21.572725v1 (2023).

Figure. S1.

**
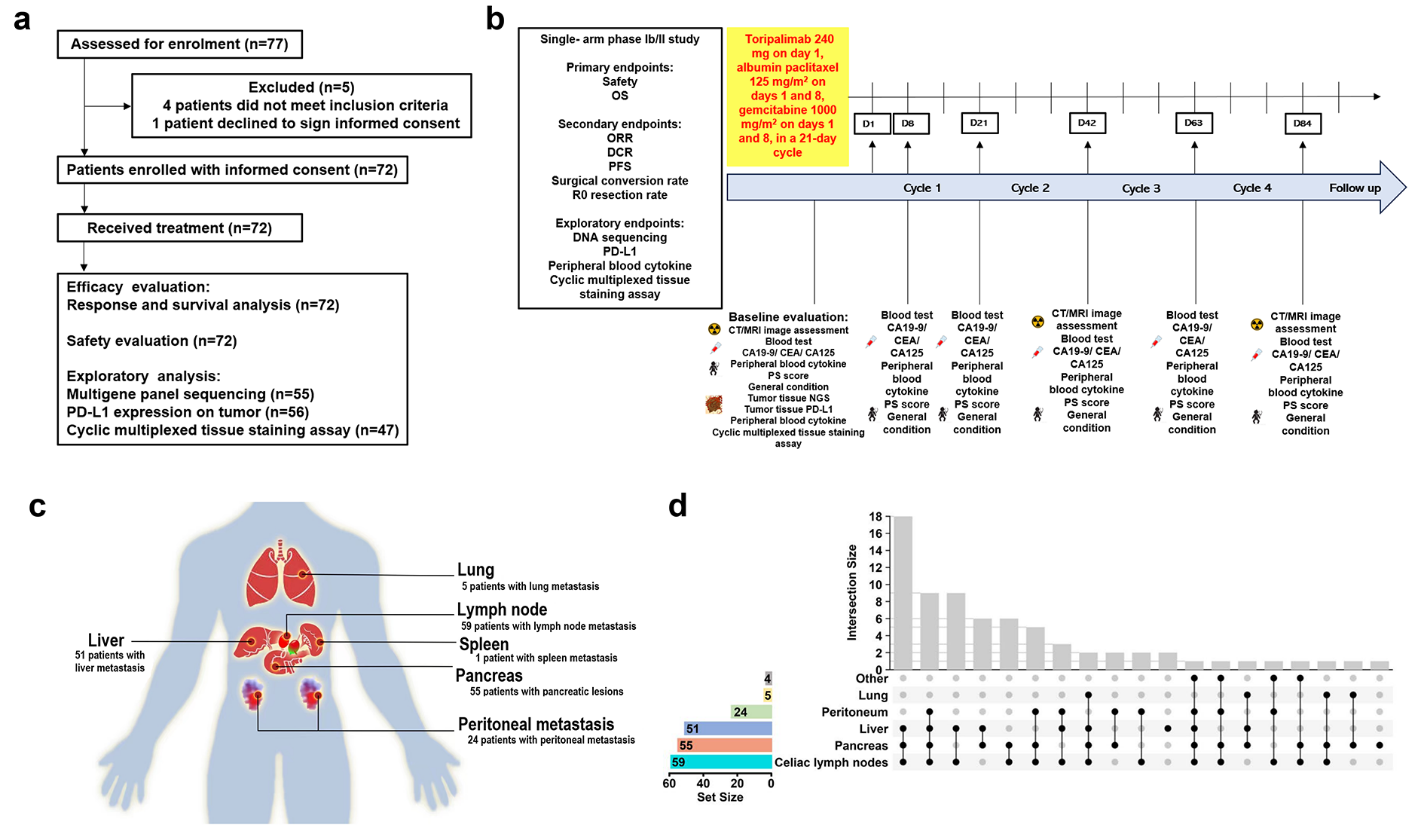
**

**Figure S1. Diagram of trial profile and patient tumor characteristics**

(a) Seventy-seven participants were assessed for eligibility, and 72 were enrolled in this study. Treatment efficacy was evaluated by response and survival (full analysis set, n=72). Safety evaluation was performed (n=72). Exploratory biomarkers analysis was performed by multigene panel sequencing (n=55), PD-L1 expression (n=56) and tumor microenvironment using cyclic multiplexed tissue staining assay (n=47).

(b) Diagram of the timeline show the treatment, efficacy, safety and sample collection protocol. The combination therapy with toripalimab and GnP was stopped until progressive disease or unacceptable adverse events, and tumor tissue samples were collected before the treatment for DNA sequencing, PD-L1 expression, and cyclic multiplexed tissue staining assay analysis. Peripheral blood samples were collected before and after the treatment for cytokines analysis.

(c) The sites with the highest frequencies of metastasis are illustrated. The celiac lymph nodes (n=59, 81.9%) and the liver (n=51, 70.8%) were most common metastatic sites.

(d) Schematic diagram of tumor burden for each patient. Forty-three (59.7%) patients had tumors at 3 or more sites. The sites of the highest frequencies of metastasis were the celiac lymph nodes (n=59, 81.9%) and the liver (n=51, 70.8%).

Figure. S2.


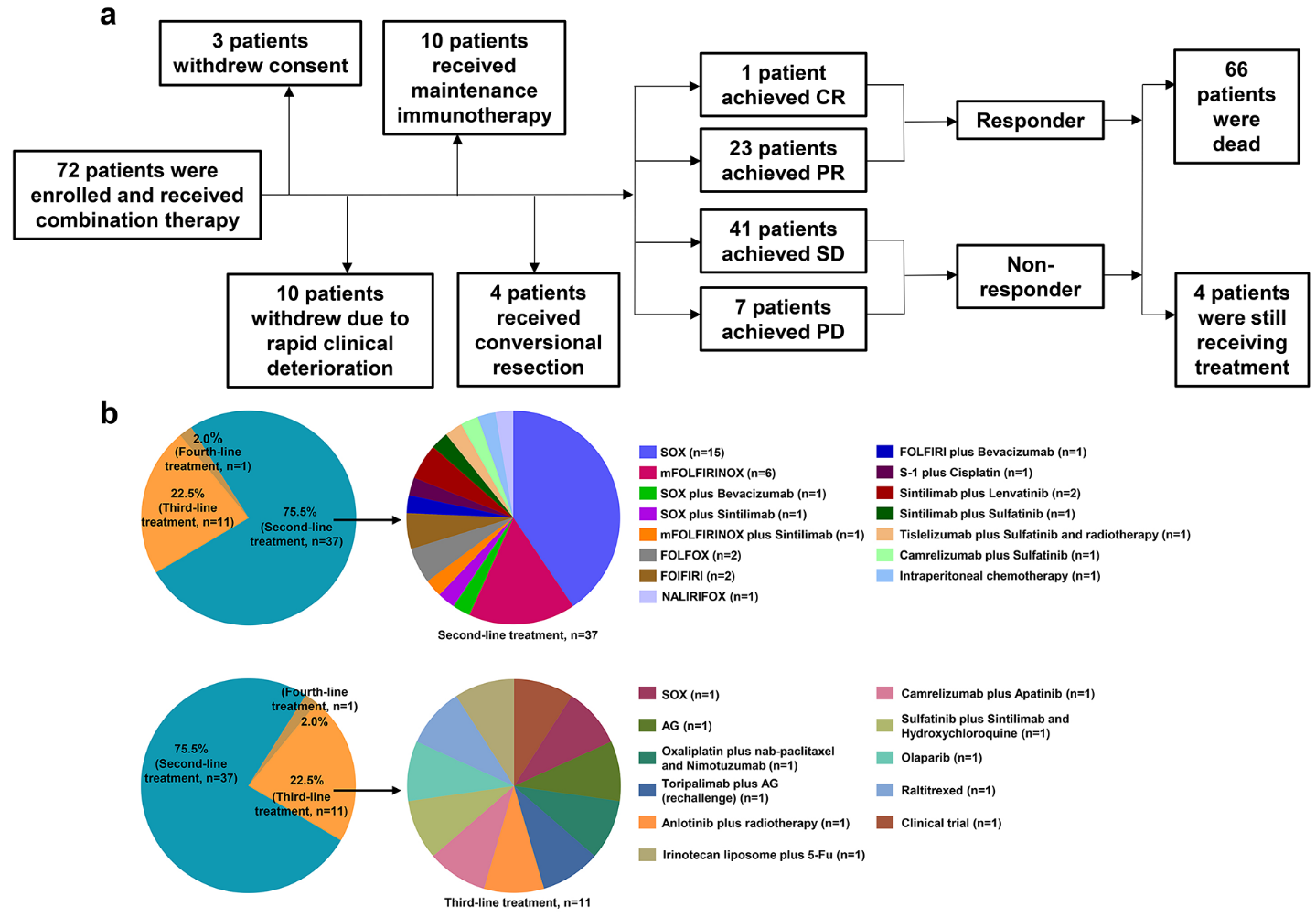


**Figure S2. Treatment and follow-up flow diagram and subsequent treatment after first-line therapy**

(a) The treatment and follow-up flow diagram displays that 66 patients were dead and 4 patients were still receiving treatment at the last follow-up.

(b) As of the study cut-off time, 45 patients had started follow-up anti-tumor therapy. Of these 45 patients, 34 (75.6%) received second-line therapy, 10 (22.2%) received third-line therapy, and 1 (2.2%) received fourth-line therapy.

Figure. S3.


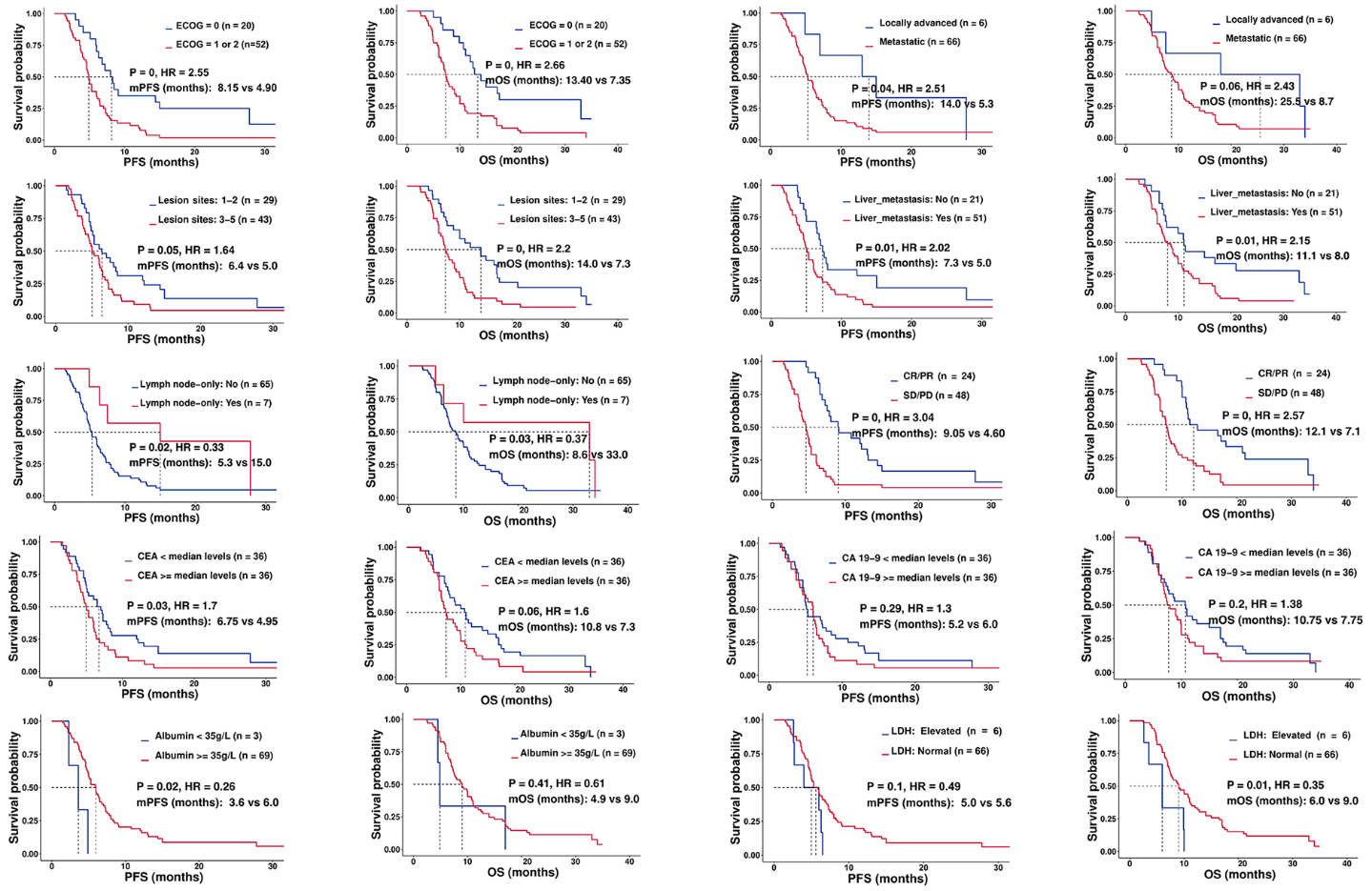


**Figure S3. Subgroup analysis based on clinical characteristics**

Progression-free survival and overall survival analysis for patients with different clinical characteristics. Log-rank test was used for curve comparison.

Figure. S4.

**
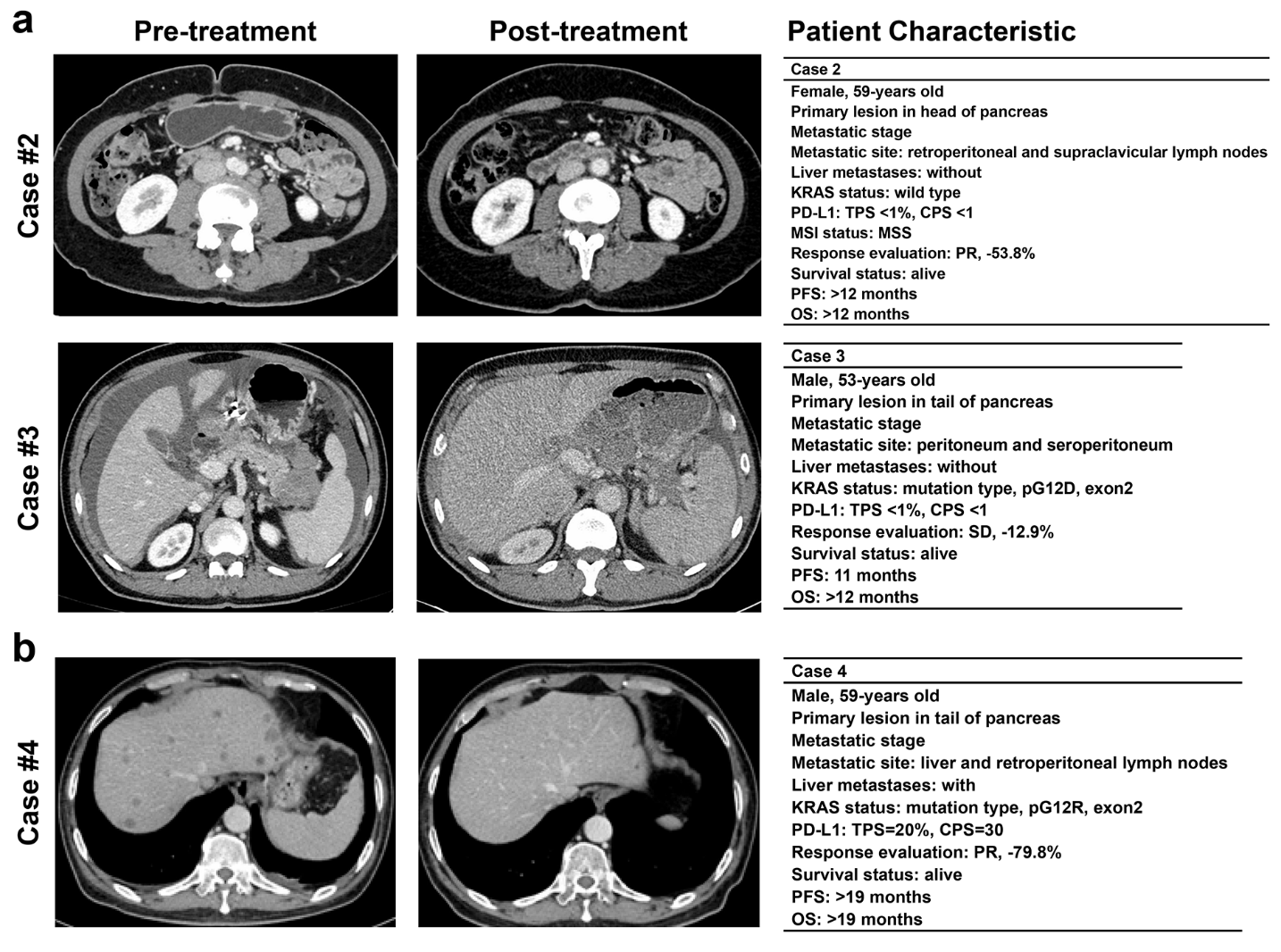
**

**Figure S4. Representative cases with non-liver metastasis status or high PD-L1 expression**

(a) Two representative PDAC cases without liver metastasis.

(b) One representative PDAC case with liver metastasis and high PD-L1 expression.

Figure. S5.


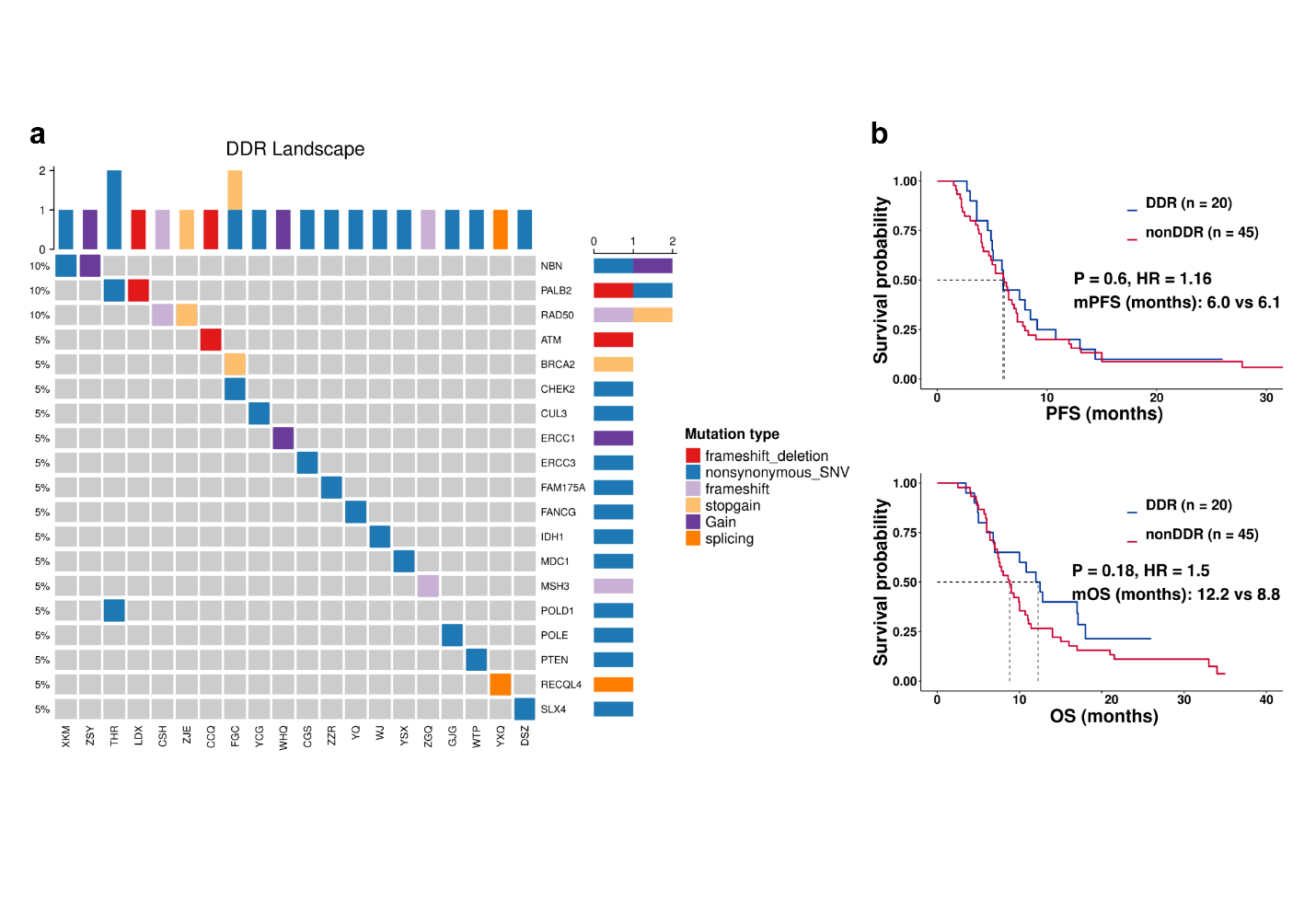


**Figure S5. DNA damage repair (DDR) features and association with patient survival**

(a) DDR-related genetic alterations identified by whole exome sequencing (WES) analysis.

(b) Progression-free survival and overall survival curves for patients with and without DDR mutation. Log-rank test was used for curve comparison.

Figure. S6.


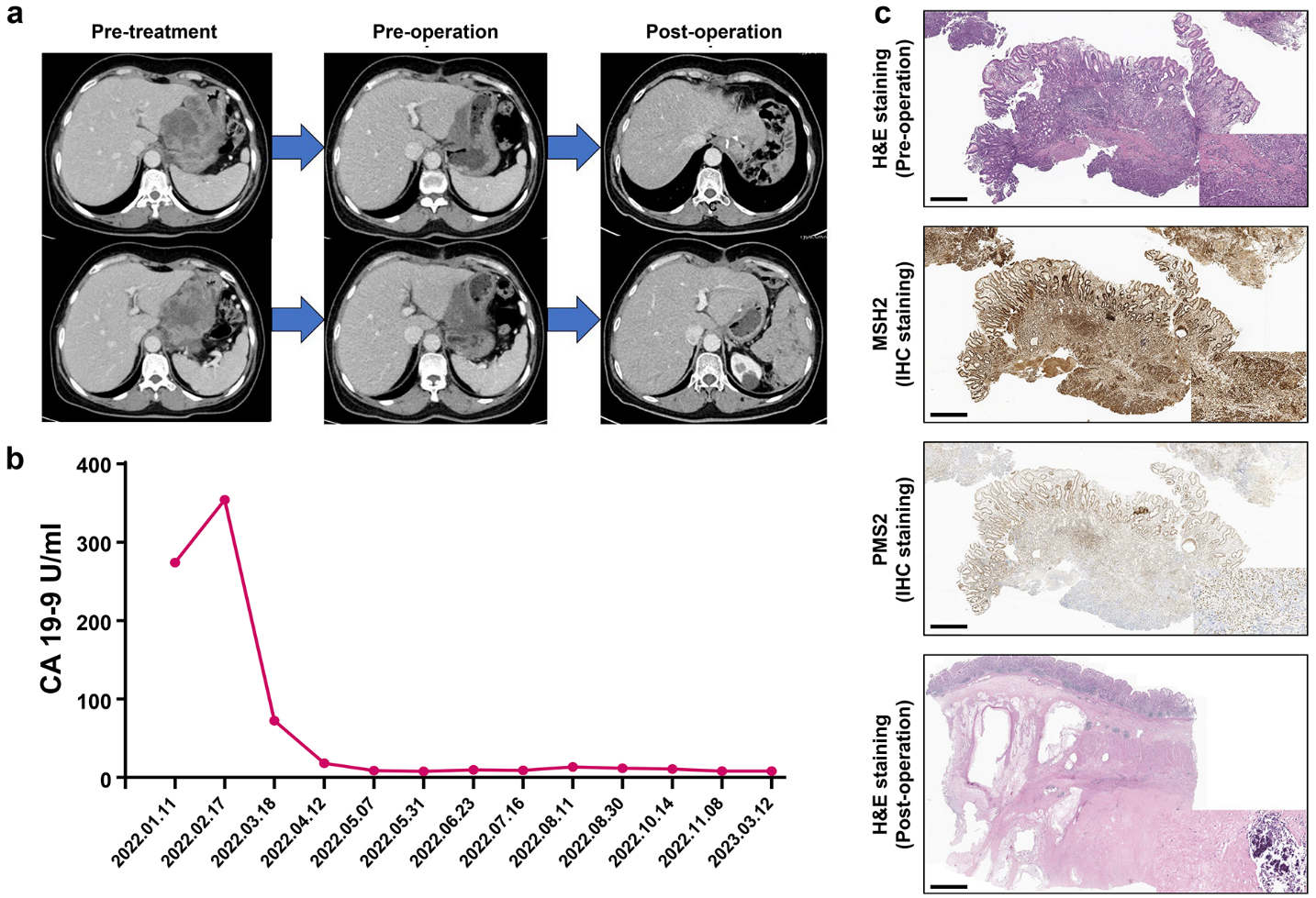


**Figure S6. Representative case with MSI-H**

(a) A patient with locally advanced pancreatic cancer with MSI-H experienced complete resection (R0) and complete response (CR) after six cycles of therapy with toripalimab combined with AG, followed by maintenance treatment with toripalimab. After successful conversion resection, CT showed no evidence of residual tumor.

(b) CA199 levels of this patient during treatment.

(c) c1, Hematoxylin and eosin (H&E) staining showed invasive pancreatic cancer cells in the stomach mucosa or submucosa; c2, immunohistochemical (IHC) staining showed MSH2-positive expression in tumor cells; c3, IHC staining showed PMS2-negative expression in tumor cells; and c4, pathological examination confirmed pathologic CR (pCR) after surgery. Scale bar: 625 µm.

Figure. S7.


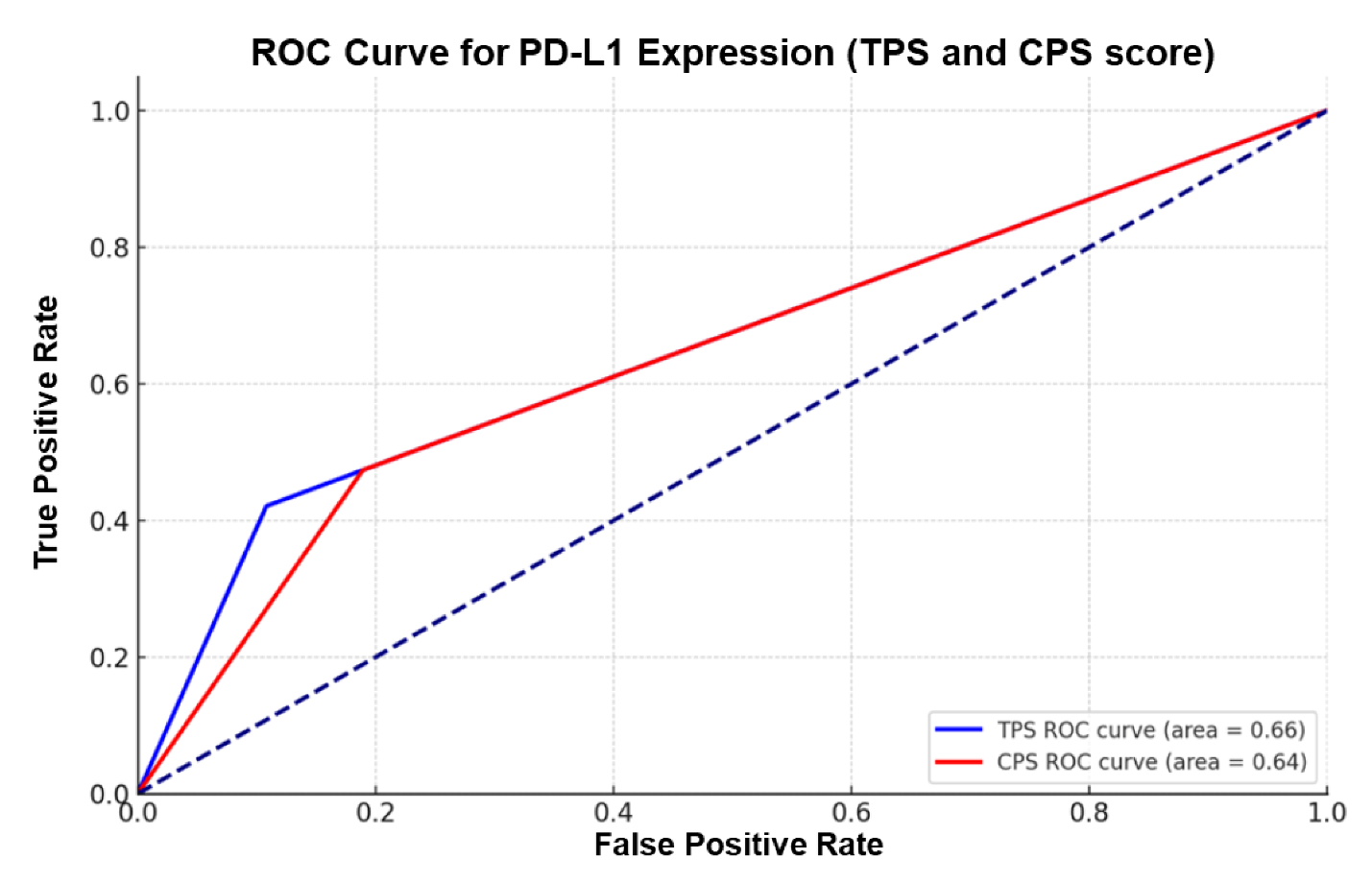


**Figure S7. ROC curve for the association between PD-L1 expression (TPS and CPS score) and OS**

The AUC value was 0.66 for the association between PD-L1 expression TPS score and OS and 0.64 for the association between PD-L1 expression CPS score and OS.

Figure. S8.


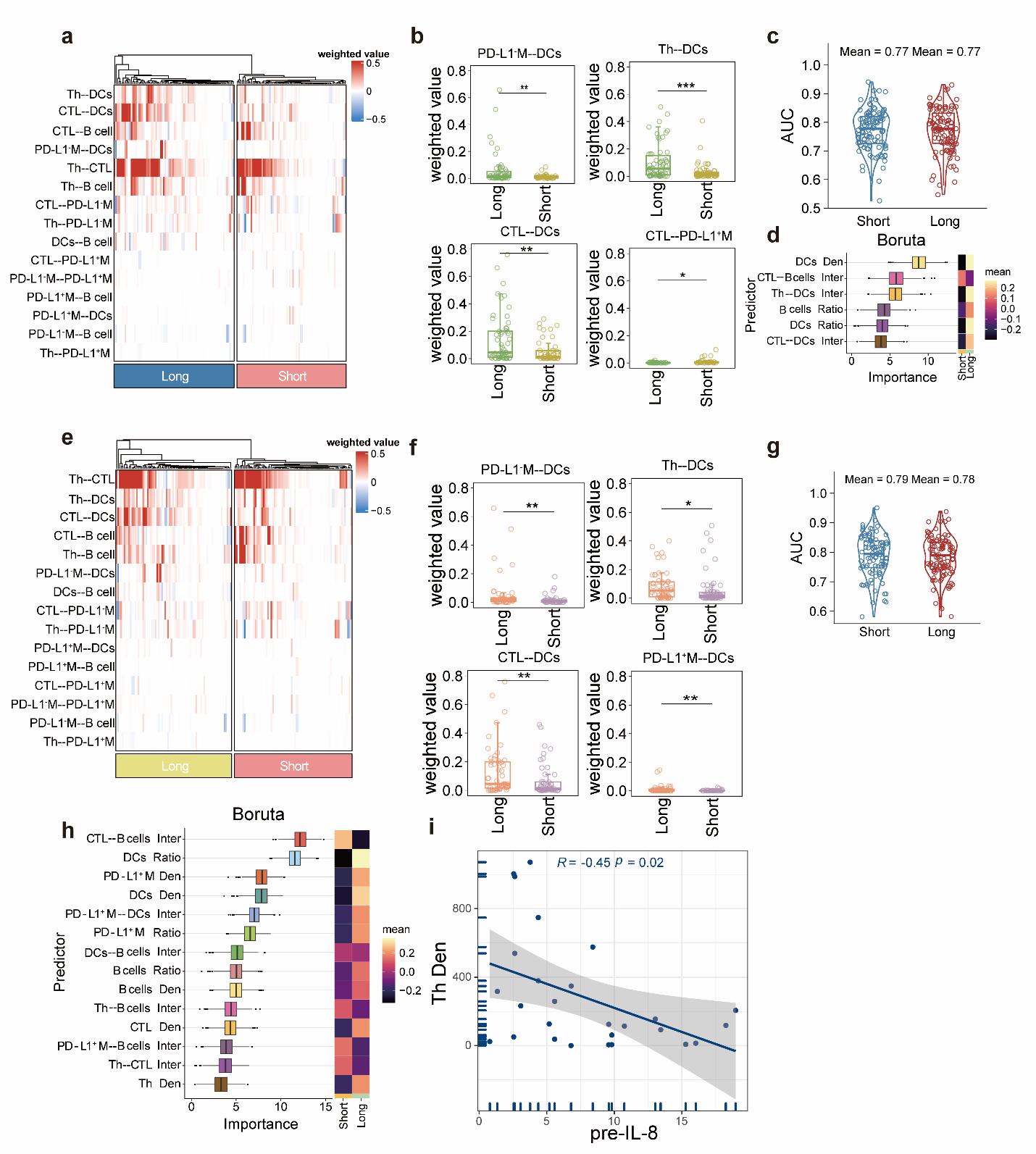


**Figure S8. Immune Cell Interactions as Predictors of Long Overall Survival and Progression-Free Survival**

1. Heatmap of cell-cell interaction intensities in patients with long (blue, n=24) and short (pink, n=23) overall survival (OS) (n=47).
2. Box plots comparing interaction intensities between long and short OS groups for key cell pairs. Significant interactions are noted for PD-L1^−^ M--DCs (*P* <0.01), Th--DCs (*P* <0.001), CTL--DCs (*P* <0.01), and CTL--PD-L1^+^ M (*P* <0.05), with higher interaction scores in long OS patients. Data are represented as mean ± SEM.
3. Violin plot showing AUC values for predictive models, demonstrating comparable predictive performance for both long and short OS groups, with a mean AUC of 0.77.
4. Boruta feature importance analysis identifying key predictors of OS.
5. Heatmap of cell-cell interaction intensities comparing long (green, n=24) and short (pink, n=23) progression-free survival (PFS) groups.
6. Box plots comparing interactions between long and short PFS groups, with significant interactions for PD-L1^−^ M--DCs (*P* <0.01), Th--DCs (*P* <0.05), CTL--DCs (*P* <0.01), and PD-L^+^ M--CTL (*P* <0.01). Data are represented as mean ± SEM.
7. Violin plot of AUC values for predictive models for PFS, with a mean AUC of 0.79 for long PFS and 0.78 for short PFS.
8. Boruta feature importance analysis ranking the most important predictors for PFS.

(i) Scatter plot showing a significant negative correlation (r= -0.45, *P*=0.02) between pre-IL-8 levels and Th density.

Table S1. All-cause adverse events, occurring during first-line therapy (induction and maintenance phase)

| Events | Patients, n (%) | | | |
| --- | --- | --- | --- | --- |
|  | Grade1-2 | Grade3 | Grade4 | Any Grade |
| TRAEs |  |  |  |  |
| Anemia | 56 (77.8) | 8 (11.1) | 2 (2.8) | 66 (91.7) |
| Hypoproteinemia | 52 (72.2) | 3 (4.2) | 1 (1.4) | 56 (77.8) |
| AST increased | 45 (62.5) | 5 (6.9) | 0 | 50 (69.4) |
| ALT increased | 42 (58.3) | 6 (8.3) | 0 | 48 (66.7) |
| Leukopenia | 32 (44.4) | 6 (8.3) | 0 | 38 (52.8) |
| Diarrhea | 35 (48.6) | 2 (2.8) | 0 | 37 (51.4) |
| Neutropenia | 26 (36.1) | 9 (12.5) | 2 (2.8) | 37 (51.4) |
| Hyponatremia | 30 (41.7) | 2 (2.8) | 0 | 32 (44.4) |
| Anorexia | 30 (41.7) | 1 (1.4) | 0 | 31 (43.1) |
| Hypocalcemia | 28 (38.9) | 0 | 0 | 28 (38.9) |
| Thrombocytopenia | 22 (30.6) | 4 (5.6) | 1 (1.4) | 27 (37.5) |
| Nausea | 25 (34.7) | 2 (2.8) | 0 | 27 (37.5) |
| Molt | 23 (31.9) | 0 | 0 | 23 (31.9) |
| Constipation | 19 (26.4) | 2 (2.8) | 0 | 21 (29.2) |
| Hypokalemia | 15 (20.8) | 6 (8.3) | 0 | 21 (29.2) |
| Peripheral sensory neuropathy | 17 (23.6) | 3 (4.2) | 0 | 20 (27.8) |
| Fatigue | 18 (25.0) | 2 (2.8) | 0 | 20 (27.8) |
| Insomnia | 18 (25.0) | 0 | 0 | 18 (25.0) |
| Vomiting | 14 (19.4) | 2 (2.8) | 0 | 16 (22.2) |
| Fever | 15 (20.8) | 0 | 0 | 15 (20.8) |
| Abdominal distension | 14 (19.4) | 0 | 0 | 14 (19.4) |
| Blood bilirubin increased | 8 (11.1) | 5 (6.9) | 0 | 13 (18.1) |
| irAEs (possibly or definitely related) | | | | |
| Hypothyroidism | 42 (58.3) | 0 | 0 | 42 (58.3) |
| Rash | 25 (34.7) | 1 (1.4) | 0 | 26 (36.1) |
| Myocardial enzyme elevation | 6 (8.3) | 2 (2.8) | 0 | 8 (11.1) |
| Central neurotoxicity | 3 (4.2) | 1 (1.4) | 0 | 4 (5.6) |
| Interstitial pneumonia | 2 (2.8) | 1 (1.4) | 0 | 3 (4.2) |

Data were n (%). The table lists all-cause adverse events (including TRAEs and irAEs) among ITT population. No grade 5 adverse event was observed. TRAEs, treatment-related adverse events; irAEs, immune-related adverse events; AST, aspartate aminotransferase; ALT, alanine transaminase.

Table S2. Univariate and multivariate analysis of factors associated with PFS using cox proportional hazards regression

| **Characteristics** | **Progression-free survival (PFS)** | | | | |
| --- | --- | --- | --- | --- | --- |
|  | **Univariate analysis Multivariate analysis** | | | | |
|  | **Median (months)** | **HR**  **(95%CI)** | **P value** | **HR**  **(95%CI)** | **P value** |
| **ECOG performance status** |  |  | <0.01 |  | 0.28 |
| 0 | 8.2 | 1 |  | 1 |  |
| 1/2 | 4.9 | 2.55 (1.43-4.55) |  | 0.61（0.25-1.50） |  |
| **Extent of disease** |  |  | 0.04 |  | 0.61 |
| locally advanced | 14.0 | 1 |  | 1 |  |
| metastatic | 5.3 | 2.51 (1.00-6.32) |  | 1.46（0.35-6.21） |  |
| **No. of lesion sites** |  |  | 0.05 |  | 0.91 |
| 1-2 | 6.4 | 1 |  | 1 |  |
| 3-5 | 5.0 | 1.64 (0.99-2.70) |  | 0.96（0.45-2.05） |  |
| **Liver metastasis** |  |  | 0.01 |  | 0.02 |
| No | 7.3 | 1 |  | 1 |  |
| Yes | 5.0 | 2.02 (1.17-3.51) |  | 3.10（1.21-7.97） |  |
| **Lymph node-only metastasis** |  |  | 0.02 |  | 0.86 |
| No | 5.3 | 1 |  | 1 |  |
| Yes | 15.0 | 0.33 (0.13-0.84) |  | 0.86（0.15-4.88） |  |
| **No. of chemotherapy cycles** |  |  | <0.01 |  | <0.01 |
| < median | 3.7 | 1 |  | 1 |  |
| ≥ median | 8.3 | 0.09 (0.04-0.17) |  | 0.06（0.01-0.31） |  |
| **No. of immunotherapy cycles** |  |  | <0.01 |  | 0.77 |
| < median | 3.9 | 1 |  | 1 |  |
| ≥ median | 8.0 | 0.15 (0.08-0.26) |  | 1.26（0.26-6.17） |  |
| **Baseline CA199 level (U/ml)** |  |  | 0.29 |  | 0.12 |
| < median | 5.2 | 1 |  | 1 |  |
| ≥ median | 6.0 | 1.30 (0.80-2.11) |  | 1.72（0.87-3.40） |  |
| **Baseline CEA level (U/ml)** |  |  | 0.03 |  | 0.43 |
| < median | 6.8 | 1 |  | 1 |  |
| ≥ median | 5.0 | 1.70 (1.04-2.77) |  | 0.72（0.32-1.63） |  |
| **Maintenance treatment** |  |  | <0.01 |  | <0.01 |
| No | 5.0 | 1 |  | 1 |  |
| Yes | 23.7 | 0.17 (0.07-0.43) |  | 0.13（0.04-0.42） |  |
| **Baseline albumin level (g/L)** |  |  | 0.02 |  | 0.17 |
| ≤35 | 3.6 | 1 |  | 1 |  |
| >35 | 6.0 | 0.26 (0.08-0.86) |  | 0.40（0.11-1.49） |  |
| **PD-L1 expression status** |  |  | 0.65 |  | 0.49 |
| CPS<5 | 5.6 | 1 |  | 1 |  |
| CPS≥5 | 6.8 | 0.87 (0.47-1.59) |  | 0.79（0.40-1.55） |  |

Univariate factors with statistical differences were included in the multivariate analysis. Given CA199 and CEA as an important tumor marker for pancreatic cancer and PD-L1 as a potential predictor of immunotherapy efficacy, these three factors were also included in the multivariate analysis. ECOG, Eastern Cooperative Oncology Group; CA199, carbohydrate antigen 19-9; PD-L1, programmed death ligand 1.

Table S3. Univariate and multivariate analysis of factors associated with OS using cox proportional hazards regression

| **Characteristics** | **Overall Survival (OS)** | | | | | |
| --- | --- | --- | --- | --- | --- | --- |
|  | **Univariate analysis Multivariate analysis** | | | | | |
|  | **Median (months)** | | **HR**  **(95%CI)** | **P value** | **HR**  **(95%CI)** | **P value** |
| **ECOG performance status** | |  |  | <0.01 |  | 0.73 |
| 0 | | 13.4 | 1 |  | 1 |  |
| 1/2 | | 7.4 | 2.66 (1.48-4.78) |  | 0.87 (0.39-1.96) |  |
| **Extent of disease** | |  |  | 0.06 |  | 0.89 |
| locally advanced | | 25.5 | 1 |  | 1 |  |
| metastatic | | 8.7 | 2.43 (0.95-6.23) |  | 1.11 (0.26-4.65) |  |
| **No. of lesion sites** | |  |  | <0.01 |  | 0.06 |
| 1-2 | | 14.0 | 1 |  | 1 |  |
| 3-5 | | 7.3 | 2.20 (1.31-3.71) |  | 2.04 (0.97-4.27) |  |
| **Liver metastasis** | |  |  | 0.01 |  | 0.28 |
| No | | 11.1 | 1 |  | 1 |  |
| Yes | | 8.0 | 2.15 (1.19-3.86) |  | 1.57 (0.70-3.51) |  |
| **Lymph node-only metastasis** | |  |  | 0.03 |  | 0.79 |
| No | | 8.6 | 1 |  | 1 |  |
| Yes | | 33.0 | 0.37 (0.14-0.95) |  | 0.81 (0.17-3.94) |  |
| **No. of chemotherapy cycles** | |  |  | <0.01 |  | 0.23 |
| < median | | 6.0 | 1 |  | 1 |  |
| ≥ median | | 11.7 | 0.23 (0.13-0.39) |  | 0.39 (0.09-1.81) |  |
| **No. of immunotherapy cycles** | |  |  | <0.01 |  | 0.52 |
| < median | | 6.2 | 1 |  | 1 |  |
| ≥ median | | 12.2 | 0.23 (0.14-0.40) |  | 0.60 (0.12-2.85) |  |
| **Baseline CA199 level (U/ml)** | |  |  | 0.20 |  | <0.01 |
| < median | | 10.8 | 1 |  | 1 |  |
| ≥ median | | 7.8 | 1.38 (0.84-2.25) |  | 3.11 (1.47-6.57) |  |
| **Baseline CEA level (U/ml)** |  | |  | 0.06 |  | 0.15 |
| < median | 10.8 | | 1 |  | 1 |  |
| ≥ median | 7.3 | | 1.60 (0.98-2.61) |  | 0.56 (0.25-1.23) |  |
| **Maintenance treatment** |  | |  | <0.01 |  | <0.01 |
| No | 7.6 | | 1 |  | 1 |  |
| Yes | 17.1 | | 0.21 (0.08-0.53) |  | 0.20 (0.06-0.62) |  |
| **LDH** |  | |  | 0.01 |  | 0.18 |
| normal | 9.0 | | 1 |  | 1 |  |
| elevated | 6.0 | | 2.85 (1.20-6.79) |  | 2.02 (0.73-5.62) |  |
| **PD-L1 expression status** |  | |  | 0.88 |  | 0.82 |
| CPS<5 | 8.9 | | 1 |  | 1 |  |
| CPS≥5 | 10.0 | | 1.05 (0.57-1.92) |  | 1.09 (0.55-2.16) |  |

Univariate factors with statistical differences were included in the multivariate analysis. Given CA199 and CEA as important tumor markers for pancreatic cancer and PD-L1 as a potential predictor of immunotherapy efficacy, these three factors were also included in the multivariate analysis. ECOG, Eastern Cooperative Oncology Group; CA199, carbohydrate antigen 19-9; CEA. carcinoembryonic antigen; PFS, progression free survival; LDH, lactate dehydrogenase; PD-L1, programmed death ligand 1.

Table S4. Chemotherapy Drug Dose Modification Form

| Adverse Events | Grading | Dose Modification | |
| --- | --- | --- | --- |
|  |  | First occurrence | Second occurrence |
| Neutropenia | Grade 2 - 3 | Delay until neutrophils ≥ 1.5 x 109/L, maintain original dose | Delay until neutrophils ≥ 1.5 x 109/L, maintain original dose |
|  | Grade 3 with fever and body temperature ≥ 38.5℃ or Grade 4 | Delay until neutrophils ≥ 1.5 x 109/L, reduce nabpaclitaxel to 100 mg/m2, and reduce gemcitabine to 800 mg/m2 | Delay until neutrophils ≥ 1.5 x 109/L, reduce nabpaclitaxel to 75 mg/m2, and reduce gemcitabine to 600 mg/m2 |
| Thrombocytopenia | Grade 2 | Delay until platelets ≥ 75 × 109/L, maintain original dose | Delay until platelets ≥ 75 × 109/L, maintain original dose |
|  | Grade 3-4 | Delay until platelets ≥ 75 x 109/L, reduce nabpaclitaxel to 100 mg/m2, and reduce gemcitabine to 800 mg/m2 | Delay until platelets ≥ 75 x 109/L, reduce nabpaclitaxel to 75 mg/m2, and reduce gemcitabine to 600 mg/m2 |
| Diarrhoea | Grade 2 | Delay treatment until toxicity ≤ Grade 1 and maintain the original dose | Delay treatment until toxicity ≤ Grade 1 and maintain the original dose |
|  | Grade 3-4 | Delay treatment until toxicity ≤ Grade 1, decrease nabpaclitaxel to 100 mg/m2, and decrease gemcitabine to 800 mg/m2 | Delay until toxicity ≤ Grade 1, reduce nabpaclitaxel to 75 mg/m2, and reduce gemcitabine to 600 mg/m2 |
| Hepatotoxicity and other non-hematological toxicities | Grade 2 | Delay until toxicity ≤ Grade 1 | Delay treatment until toxicity ≤ Grade 1, decrease nabpaclitaxel to 100 mg/m2, and decrease gemcitabine to 800 mg/m2 |
|  | Grade 3 | Delay treatment until toxicity ≤ Grade 1, decrease nabpaclitaxel to 100 mg/m2, and decrease gemcitabine to 800 mg/m2 | Delay until toxicity ≤ Grade 1, reduce nabpaclitaxel to 75 mg/m2, and reduce gemcitabine to 600 mg/m2 |
|  | Grade 4 | Delay until toxicity ≤ Grade 1, reduce nabpaclitaxel to 75 mg/m2, and reduce gemcitabine to 600 mg/m2 | Drug withdrawn |
